# Feasibility of an Electronic Health Tool to Promote Physical Activity in Primary Care: Pilot Cluster Randomized Controlled Trial

**DOI:** 10.2196/15424

**Published:** 2020-02-14

**Authors:** Payal Agarwal, Natasha Kithulegoda, Zachary Bouck, Beth Bosiak, Ilana Birnbaum, Lindsay Reddeman, Liane Steiner, Liora Altman, Robin Mawson, Roni Propp, Jane Thornton, Noah Ivers

**Affiliations:** 1 Women’s College Hospital Institute for Health Systems Solutions and Virtual Care Women's College Hospital Toronto, ON Canada; 2 Department of Family and Community Medicine University of Toronto Toronto, ON Canada; 3 St Michael's Hospital Toronto, ON Canada; 4 Ontario Ministry of Health and Long-Term Care Toronto, ON Canada; 5 Rehabilitation Sciences Institute University of Toronto Toronto, ON Canada; 6 Fowler Kennedy Sport Medicine Clinic Western University London, ON Canada

**Keywords:** eHealth, primary care, physical activity, patient-centered care

## Abstract

**Background:**

Physical inactivity is associated with increased health risks. Primary care providers (PCPs) are well positioned to support increased physical activity (PA) levels through screening and provision of PA prescriptions. However, PCP counseling on PA is not common.

**Objective:**

This study aimed to assess the feasibility of implementing an electronic health (eHealth) tool to support PA counseling by PCPs and estimate intervention effectiveness on patients’ PA levels.

**Methods:**

A pragmatic pilot study was conducted using a stepped wedge cluster randomized trial design. The study was conducted at a single primary care clinic, with 4 pre-existing PCP teams. Adult patients who had a periodic health review (PHR) scheduled during the study period were invited to participate. The eHealth tool involved an electronic survey sent to participants before their PHR via an email or a tablet; data were used to automatically produce tailored resources and a PA prescription in the electronic medical record of participants in the intervention arm. Participants assigned to the control arm received usual care from their PCP. Feasibility was assessed by the proportion of completed surveys and patient-reported acceptability and fidelity measures. The primary effectiveness outcome was patient-reported PA at 4 months post-PHR, measured as metabolic equivalent of task (MET) minutes per week. Secondary outcomes assessed determinants of PA, including self-efficacy and intention to change based on the Health Action Process Approach behavior change theory.

**Results:**

A total of 1028 patients receiving care from 34 PCPs were invited to participate and 530 (51.55%) consented (intervention [n=296] and control [n=234]). Of the participants who completed a process evaluation, almost half (88/178, 49.4%) stated they received a PA prescription, with only 42 receiving the full intervention including tailored resources from their PCP. A cluster-level linear regression analysis yielded a non–statistically significant positive difference in MET-minutes reported per week at follow-up between intervention and control conditions (mean difference 1027; 95% CI −155 to 2209; *P*=.09). No statistically significant differences were observed for secondary outcomes.

**Conclusions:**

Our results suggest that it is feasible to build an eHealth tool that screens and provides tailored resources for PA in a primary care setting but suboptimal intervention fidelity suggests greater work must be done to address PCP barriers to resource distribution. Participant responses to the primary effectiveness outcome (MET-minutes) were highly variable, reflecting a need for more robust measures of PA in future trials to address limitations in patient-reported data.

**Trial Registration:**

ClinicalTrials.gov NCT03181295; https://clinicaltrials.gov/ct2/show/NCT03181295

## Introduction

### Background

Physical inactivity is the fourth leading risk factor for global morbidity and mortality, responsible for 6% of deaths annually [1]. The Canadian Physical Activity Guidelines recommend at least 150 min of moderate-to-vigorous activity per week for adults aged 18 to 64 years [2]. In those who achieve recommended levels of physical activity (PA), all-cause mortality is decreased by 19% to 30% [3,4], with a dose response identified [5,6]. Despite this evidence, it is estimated that only 16% of adults aged 18 to 79 years in Canada meet current PA guidelines [7].

Primary care physicians (PCPs) are ideally positioned to positively affect levels of PA among their patients [8]. Multiple clinical guidelines recommend PCPs screen patients for current activity levels and offer targeted counseling during routine visits [9-12]. Evidence indicates that a tailored PA prescription from PCPs can improve overall activity levels [13-15]. Unfortunately, this is rarely implemented in real-world clinical practice [16-19], with reported barriers including lack of time, knowledge, and training in PA counseling and a perceived inability to change patient behavior [20,21].

Electronic screening of health behaviors can save time for PCPs and has been highly accepted by patients as a method to share information with their care team [22-24], and there is evidence supporting the use of digital health tools to improve PA [25]. Furthermore, using computers to deliver tailored messaging and resources to patients can have a positive impact on behavior change, including PA, relative to more traditional methods of health counseling [26-31]. Integrating screening and tailored information provision into one intervention may help change PA levels by addressing the complex needs of both providers and patients [24,26].

### Objective

In this pilot study, we tested the feasibility of implementing an electronic health (eHealth) tool to support PA counseling in routine primary care and produced a preliminary estimate of intervention effectiveness on changing PA levels. Our aim was to optimize the intervention, evaluate recruitment and retention of participants, and assess suitability of the primary outcome for a subsequent, larger definitive trial [32,33].

## Methods

### Design

This pilot study has been reported in accordance with extensions to the Consolidated Standards of Reporting Trials 2010 statement for both randomized pilot studies [33] and stepped wedge cluster randomized trials (SW-CRT) [34] and the standards for reporting implementation studies statement [35]. Research ethics approval was obtained from the Women's College Hospital Research Ethics Board (registered on ClinicalTrials.gov as NCT03181295).

We conducted a pilot study using a pragmatic SW-CRT design to identify potential issues with implementation or analysis that might challenge the feasibility of future trials involving more clusters [36]. PCP teams functioning as naturally occurring clusters of clinicians and patients were randomized to allow gradual implementation of the tool and prevent intervention contamination across clusters [37-39]. The study was divided into 5 periods, each one 6 weeks in length. Initially, no teams were exposed to the intervention [38], then 1 team was randomly assigned to begin the intervention at the start of each period [37]. Randomization occurred using computer-generated random numbers produced by an independent statistician [39]. Participants and researchers could not be blinded due to the nature of the intervention.

### Setting

The study was conducted at the Women’s College Hospital Family Practice Health Centre (FPHC), an academic, multidisciplinary family health team located in Toronto, Ontario, Canada, between February 20, 2017, and March 17, 2018. The FPHC has 39 PCPs and over 50,000 clinical visits per annum. The FPHC is divided into 4 teams for operational convenience with minimal clinician or patient crossover among teams.

### Participants and Recruitment

All PCPs (N=39) at the FPHC were eligible to participate in the study, exempting learners and PCPs who were not expected to be present for the entire study period. Patients rostered to a participating PCP were eligible if they attended a periodic health review (PHR) during the study period and were aged 18 to 79 years at the time of the PHR. PHRs were considered appropriate opportunities to deliver the intervention, as they focus on preventative care counseling [40]. Patients deemed unable to safely or effectively complete the intervention at the time of their PHR were excluded. This included those with dementia or cognitive impairment, with major active illness, or who were pregnant. Non-English speakers were also excluded because of a lack of resources to appropriately accommodate other languages. A research assistant was responsible for regularly reviewing the FPHC schedule and assessing potential eligibility via electronic medical record (EMR) review. The PCP confirmed eligibility when the research assistant was uncertain.

### Intervention Development

The Health Action Process Approach (HAPA) is a theory of behavior change used to inform the design of successful behavior change interventions, including those targeting PA [41]. It aligns with factors such as goal-setting, which has been shown to improve PA in some digital health interventions [42]. In general, HAPA suggests that individuals who have not yet developed an intention to change behavior (*preintenders*) may benefit from interventions that target risk perception and outcome expectancy. Those who have developed an intention to modify behaviors but have not yet shown change (*intenders*) may benefit from interventions that target action planning and coping planning. Those who have achieved certain health behaviors (*actors*) may benefit from interventions focused on relapse prevention. Movement through these phases is fluid, affected by social support and/or contextual barriers, and is mediated by self-efficacy in action, maintenance, and recovery [41]. This approach was used to categorize participants, customize intervention materials for each participant, and analyze outcomes as described further on.

The intervention was refined using principles of user-centered design. This approach emphasizes the use of iterative product design with ongoing feedback from the end user to drive improvements and optimize the acceptance and use of the tool [43-46]. This involved multiple interviews with potential end users, as described in another paper [47].

### Treatment Group: Intervention

All patients deemed eligible for the study received an email 2 weeks before their visit with a link to a secure electronic survey (e-survey). Those who did not complete the survey before their appointment were approached in the clinic, and the e-survey was completed using a digital tablet in the waiting room. The e-survey collected informed consent, assessed baseline PA, and assessed perceived barriers and motivators for PA.

The intervention included 3 key components that were automatically generated based on the baseline survey. First, responses were summarized in the patient’s EMR along with a statement comparing the results with current PA guidelines of 150 min of moderate-to-vigorous PA per week [12,48,49]. Second, the EMR was populated with a link to 1 out of 5 toolkits that included online and community-based resources tailored to the patient’s current PA levels and perceived barriers, and an additional condition-specific PA toolkit if the patient reported any other condition (eg, cardiovascular disease). Third, a customized PA prescription was generated based on current PA levels and patient-identified motivators to increase PA. During the PHR, the prescription could be edited by the PCP based on discussions with the patient and then printed along with the toolkit for the patient to take home. Each patient’s toolkit was also sent to them 2 weeks after the PHR via mail or email. A full description and examples of the prescription and toolkit can be found in Multimedia Appendix 1 [50].

To encourage intervention fidelity, one of the principal investigators (PA or NI) spoke with each of their PCP colleagues for 5 to 15 min before their cluster switching to the intervention arm. The intervention, including EMR outputs, was demonstrated using a *test* patient chart in the EMR, and then a handout was reviewed, which addressed both workflow integration and evidence for PA counseling (see Multimedia Appendix 2 for the handout).

### Control Group: Usual Care

Participants in the usual care group completed the same baseline questionnaire as the intervention group, but no EMR outputs or patient toolkits were produced. Participating PCPs were encouraged to provide PA advice (or not) as per their normal routines, for example, no attempt was made to standardize usual care. PCPs received education about the intervention only in the week before the intervention being activated for their team.

### Outcomes and Data Collection

After exposure to the intervention, every intervention participant received a paper survey immediately after their appointment, or an e-survey 1 day following their appointment if they did not complete the paper version, to collect process measures (see Multimedia Appendix 3). Acceptability was measured using a 5-point Likert scale ranging from *very dissatisfied* to *very satisfied*. Participants were also asked about the number of min of PA counseling they received (no discussion, less than 2 min, 2-5 min, 5-10 min, or more than 10 min) and if they received a PA prescription (yes/no). Feasibility was also assessed in part by the number of eligible patients who completed a baseline survey and the frequency of missing or inaccurate data [36].

The primary effectiveness outcome was patient-reported PA at 4 months post-PHR, measured as metabolic equivalent of task (MET) minutes per week using the international physical activity questionnaire-short form (IPAQ-SF) [51]. The IPAQ-SF was selected for its short length, ease of administration, good test-retest reliability, and low cost [51].

Secondary outcomes, also collected 4 months post-PHR, assessed attitudes toward PA using the HAPA constructs to guide the assessment of proximal changes [52]. Specifically, 3 subdimensions of self-efficacy (action, recovery, and maintenance) were assessed, each measured via 2 questions (using a 4-point Likert scale ranging from *strongly disagree* to *strongly agree*) [53-55]. A score for each subdimension of self-efficacy was calculated by summing the 2 questions, dividing by the maximum possible score and multiplying by 100 (for self-efficacy scores ranging from 0-100). The total self-efficacy score was the average of all subdimension scores.

Participants’ intention regarding PA was measured in a 2-step process. Those meeting recommended PA guidelines of 150 min of moderate to vigorous activity a week were defined as *actors* [2]. Participants not meeting the recommended guidelines were defined as *nonactors*. This group was further subdivided into *intenders* and *preintenders*. Those who agreed with the statement, “I have made the decision to take part in a new kind of physical activity or increase my amount or intensity of physical activity soon” were deemed to be *intenders*, while those who disagreed were labeled *preintenders*. An e-survey collected responses for both primary and secondary outcomes and data were securely transferred and collated into a single, study-specific database (see Multimedia Appendix 4 for the survey).

### Statistical Analysis

Analysis of the pilot data was mainly descriptive [36]. The distribution of patient- and PCP-level baseline characteristics were summarized by team via means and standard deviations (median and interquartile range when skewed) and frequencies and proportions, respectively [38,39].

#### Feasibility

To understand the feasibility of our study protocol, the frequency and proportion of patients that were assessed as eligible, recruited, randomized, and who had responded to both baseline and follow-up surveys were independently summarized. Additionally, responses to the process evaluation survey were summarized by patients exposed to the intervention—via counts and proportions for categorical responses and means and standard deviations for continuous responses—to elucidate patient satisfaction with their PA counseling and patient-reported impressions of PCP acceptability and adherence to the intervention. We assessed significant differences between those who received and those who did not receive the intervention using a chi-square test of independence.

#### Preliminary Effectiveness

The presence of few clusters in our study limits options for estimating the preliminary effectiveness of the intervention. Specifically, it precludes the use of conventional analytic approaches for stepped wedge trials [34,37,38] that model patient-level responses while accounting for clustering via random effects, which require observations on many clusters to yield unbiased estimates and accurate standard errors [56,57]. Correspondingly, patient-level responses to the primary outcome (measured in MET-minutes per week) were aggregated to the cluster-period level, removing the need to adjust for patient- or PCP-level characteristics or clustering of patient responses within teams [58]. To obtain a preliminary estimate of intervention effectiveness on the primary outcome, the cluster-period mean response was then regressed as the outcome using linear regression with intervention exposure as the primary independent variable and the following fixed effects included as covariates: team (cluster), period, and mean baseline (or pretest) value [58]. To assess the robustness of our findings to statistical outliers, a sensitivity analysis was conducted involving regression analysis as specified for the primary outcome; however, before aggregating patient-level responses to the cluster-period level, those patient responses in the top 5% by primary outcome value were excluded.

Secondary outcomes were analyzed similarly. Preliminary treatment effect estimates on each self-efficacy measure (action, recovery, maintenance, and overall) were obtained using multivariable linear regression with the unit of analysis as the cluster-period and adjustment for team, period, and baseline response as covariates. With regard to intention to change PA levels, the proportion of participants meeting criteria as an *actor* or *intender* at follow-up was calculated per cluster-period and expressed as a percentage. This value was then regressed as the outcome in a negative binomial regression model with intervention exposure as the primary independent variable, adjusting for period, team, and for the proportion meeting the outcome at baseline. A similar model focused only on the proportion of participants meeting criteria as an *actor*.

For each primary and secondary outcome, the analysis was limited to patients who were randomized, attended their PHR, and provided baseline and follow-up data for that outcome. Statistical significance was assessed, where applicable, using a two-sided *P* value of .05. SAS 9.4 (SAS Institute) was used to perform all analyses.

#### Sample Size Calculation

Following standard calculations for stepped wedge trials [59], assuming an average of 30 patients per cluster period for 5 periods total (4 steps), an estimated intracluster coefficient of 0.05 and cluster autocorrelation of 0.8, significance level at 5%, and assuming a standard deviation of 300 MET-min and a conservative loss to follow-up of 20%, we would have 80% power to observe a mean difference (MD) of 150 MET-min (over the past week) between the intervention and control condition [60,61]. This corresponds to recruiting a total of 440 patients across all periods. To maximize our ability to recruit the necessary amount in each period, the time interval for each period was set at 6 weeks.

## Results

### Participants and Recruitment

**Figure 1:**
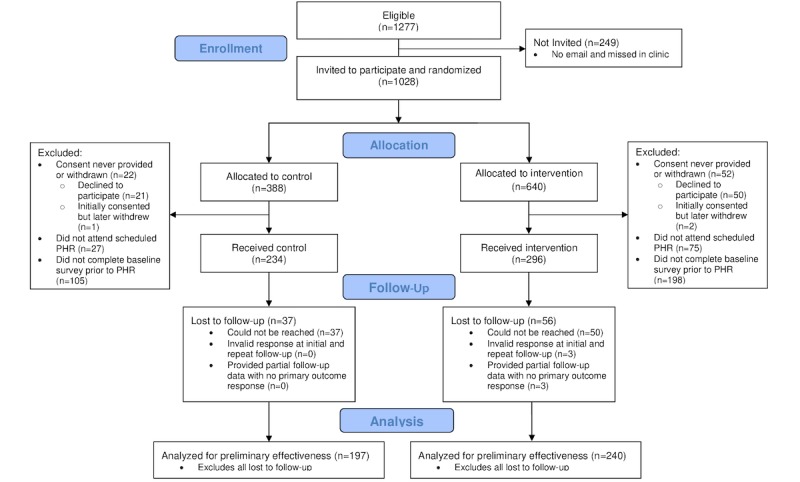
Consolidated Standards of Reporting Trials flow diagram. PHR: periodic health review.

In total, 34 out of 39 eligible PCPs participated across the 4 teams in the clinic. Of 1277 eligible patients, 1028 were invited to participate and 948 consented. Randomization proceeded based on cluster allocation (see Figure 1). In total, 46.3% (296/640) and 60.3% (234/388) individuals randomized to the intervention and control groups, respectively, completed the baseline survey and received their allocated treatment. Most participants (307/530, 57.9%) completed the baseline survey via email before their PHR; 15.1% (80/530) participants completed the survey via a tablet (because no email address was informed in their EMR) and 27.0% (143/530) completed the survey via a tablet (after being sent the survey via an email).

Table 1 describes baseline patient- and PCP-level characteristics within the 4 clusters. In terms of PCP characteristics, teams 1 and 4 were largely composed of female PCPs, and team 4 had a substantially lower median number of years since graduation. Most patient-level characteristics were balanced among teams, including baseline PA levels; patients in teams 2 and 4 reported a greater number of barriers to PA and a larger proportion of patients in team 1 were deemed actors at baseline, particularly when compared with patients in team 3 or 4. Maintenance self-efficacy was the measure with the highest degree of missingness (50/530, 9.4% missing).

**Table 1:** Primary care providers- and patient-level characteristics at baseline by team.

Characteristic				Team				Overall	
				1	2	3	4		
**PCP^a^ level**									
	PCPs, n			8	9	8	9		34
	Female, n (%)			8 (100)	6 (66)	5 (62)	8 (88)		27 (79)
	Years since graduation, median (Q1, Q3)			18 (14, 28)	26 (0, 32)	30 (7, 30)	8 (2, 16)		15 (4, 30)
**Patient level**									
	Patients with baseline data, n			131	143	118	138		530
	Age (years), mean (SD)			52 (13.3)	50 (14.8)	52 (12.7)	53 (12.8)		52 (13.4)
	Female, n (%)			110 (84.0)	95 (66.4)	80 (67.8)	122 (88.4)		407 (76.8)
	Total MET^b^-min, median (Q1, Q3)			2502 (1453, 5028)	2866 (1499, 5172)	2974 (1273, 4973)	2601 (1634, 4986)		2768 (1453, 5028)
	Total MET-min, mean (SD)			3719 (3466)	4242 (4586)	3811 (3818)	3672 (3293)		3868 (3832)
	Cardiovascular disease, n (%)			17 (13.0)	19 (13.3)	15 (12.7)	24 (17.4)		75 (14.2)
	Respiratory disease, n (%)			7 (5.3)	9 (6.3)	6 (5.1)	13 (9.4)		35 (6.6)
	Diabetes, n (%)			3 (2.3)	7 (4.9)	3 (2.5)	9 (6.5)		22 (4.2)
	Mental health issues, n (%)			25 (19.1)	26 (18.2)	19 (15.1)	19 (13.8)		89 (16.8)
	Musculoskeletal disorder, n (%)			26 (19.9)	32 (22.4)	28 (23.7)	38 (27.5)		124 (23.4)
	Neurological disorder, n (%)			1 (0.7)	3 (2.1)	3 (2.5)	2 (1.5)		9 (1.7)
	Cancer, n (%)			7 (5.3)	8 (5.6)	8 (6.8)	3 (2.2)		26 (4.9)
	No history of above diseases, n (%)			65 (49.6)	69 (48.3)	56 (47.5)	62 (44.9)		252 (47.5)
	Number of motivators, mean (SD)			5.69 (2.30)	5.50 (2.41)	6.02 (2.18)	5.36 (2.44)		5.62 (2.38)
	Number of barriers, mean (SD)			0.94 (1.23)	1.14 (1.32)	0.88 (1.16)	1.28 (1.28)		1.07 (1.26)
**Behavior change category based on HAPA^c^ theory of behavior change**									
	Actor, n (%)			80 (61.5)	69 (48.6)	32 (27.1)	35 (25.4)	216 (40.8)	
	Intender, n (%)			32 (24.6)	52 (36.6)	57 (48.3)	61 (44.2)	202 (38.1)	
	Preintender, n (%)			18 (13.9)	21 (14.8)	29 (24.6)	42 (30.4)	110 (20.8)	
	Missing, n			1	1	0	0	2 (0.4)	
**Task self-efficacy, mean (SD)**				79.3 (16)	79.3 (15)	79.2 (14.2)	79.8 (17.9)	79.4 (15.8)	
	Missing, n			10	3	9	11	33	
**Maintenance self-efficacy, mean (SD)**				80.8 (15.3)	80.3 (14.3)	80.7 (15.5)	81.8 (15.4)	80.9 (15.1)	
	Missing, n			16	19	13	12	60	
**Recovery self-efficacy, mean (SD)**				82.3 (13.9)	82.1 (13.9)	83 (13)	81.3 (15.1)	82.1 (14)	
	Missing, n			3	2	2	4	11	
**Overall self-efficacy, mean (SD)**				80.7 (13.2)	80.4 (12.7)	80.8 (12.3)	80.7 (14.6)	80.7 (13.2)	
	Missing, n			4	3	5	6	18	

^a^PCP: primary care provider.

^b^MET: metabolic equivalent of task.

^c^HAPA: Health Action Process Approach.

### Feasibility Evaluation

In total, 61.8% (183/296) patients exposed to the intervention handed in a process evaluation survey following their PHR, with 63.2% (112/183) completing fully. Overall, fewer than half of respondents (88/178, 49.4%) stated they received at least a PA prescription from their PCP. A chi-square test of independence indicated no significant difference in the proportion of patients who received at least a PA prescription versus no materials between teams (χ^2^_3_=3.0; *P*=.39). Among the 88 patients who received a PA prescription, just under half (42/88, 47%) also received tailored resources to take home. The proportion of intervention patients who completed the process evaluation receiving both a PA prescription and resources ranged from a low of 9% (6/64 patients) for team 1 to a high of 45% (15/33 patients) for team 3.

Only 6.6% (12/183) patients completing a process evaluation indicated that no PA discussion occurred during their appointment. Nearly half (86/176, 48.9%) of the participants who estimated the length of their PA discussion reported a length of 2 to 5 min, and patients in team 4 were more likely to report a talk of less than 2 min. Most patients reported being satisfied with their PA discussion irrespective of team, with no patients indicating they were dissatisfied. Of the process evaluation questions, patient satisfaction with their PA counseling (if applicable) was most prone to missing responses, with only 62.3% (114/183) providing a response. See Multimedia Appendix 5 for a full summary of process evaluation results.

### Preliminary Effectiveness Outcomes

The primary outcome (ie, total MET-minutes) was obtained for 82.5% (437/530) participants, with similar response rates among intervention (240/296, 81%) and control groups (197/234, 84.2%; see Multimedia Appendix 6). Several baseline characteristics, including having a respiratory disease and lower number of motivators, were associated with a patient’s odds of having a missing follow-up response for the primary outcome.

Before the preliminary effectiveness analysis, we independently aggregated patient responses at baseline (pre) and follow-up (post) to the cluster-period level. Table 2 summarizes the resulting 20 follow-up observations, each of which represents the mean number of MET-minutes per week reported among patients within a team (cluster) at a specific time (period) at follow-up. Comparing intervention with control within teams (ignoring time), a positive, albeit non–statistically significant difference in total MET-minutes per week was found at 4 months (team 1, MD 1412, 95% CI −2023 to 4846; team 2, MD 732, 95% CI −1059 to 2522; team 3, MD 292, 95% CI −1550 to 2133; and team 4, MD 1370, 95% CI −650 to 3391). After adjusting for time (period) and mean number of MET-minutes at baseline, cluster-level linear regression yielded a non–statistically significant difference in the grand mean number of MET-minutes reported per week at follow-up between intervention and control conditions (MD 1027, 95% CI −155 to 2209, *P*=.09).

A sensitivity analysis was conducted where any participants with a follow-up response in the top 5% (ie, ≥12,780 MET-min) were identified as statistical outliers. In total, 22 participants were flagged as outliers (intervention, n=14, 5%; control, n=8, 4%). Outliers were, on average, more likely to self-report a significantly greater number of MET-minutes at baseline versus nonoutliers (MD 5650, 95% CI 4082 to 7218); otherwise, the distribution of all other baseline characteristics was statistically equivalent between outliers and nonoutliers. After excluding outliers, the subsequent linear regression yielded a non–statistically significant and less positive (closer to the null) difference in the grand mean number of MET minutes reported per week between intervention and control conditions (MD 487, 95% CI −298 to 1273; *P*=.22).

There were no significant treatment effects on action self-efficacy (n=392; MD intervention-control −1.73, 95% CI −5.56 to 2.11, *P*=.38), maintenance self-efficacy (n=361; MD intervention-control −1.92, 95% CI −5.68 to 1.85, *P*=.32), recovery self-efficacy (n=420; MD intervention-control 2.28, 95% CI −1.39 to 5.94, *P*=.22), and overall self-efficacy (n=413; MD intervention-control 1.13, 95% CI −1.73 to 4.00, *P*=.44). There were also no significant differences in the mean proportion (PR) of subjects who were in the volitional phase at 4 months (PR intervention/control 0.95, 95% CI 0.14 to 6.66; *P*=.96), or those who were classified as *actors* at 4 months (PR intervention/control 0.88, 95% CI 0.11 to 7.12; *P*=.91).

**Table 2:** Preliminary effectiveness of intervention on primary outcome among complete cases.

Team	Posttest PA^a^ mean in MET^b^, minutes per week (95% CI)				
	Study period 1^c^	Study period 2^d^	Study period 3^e^	Study period 4^f^	Study period 5^g^
1	2636 (1432-3840)	3535 (2208-4861)^h^	6727 (2463-10992)^h^	3418 (2405-4431)^h^	2941 (1989-3893)^h^
2	2277 (1280-3274)	3942 (2289-5595)	3996 (2379-5613)^h^	3702 (1571-5832)^h^	4545 (2407-6682)^h^
3	4889 (2293-7484)	2774 (1597-3951)	3867 (1802-5932)	5223 (2166-8281)^h^	3365 (1993-4737)^h^
4	3918 (2220-5615)	4129 (2237-6020)	2596 (1965-3226)	4256 (1805-6708)^h^	4936 (2272-7600)^h^

^a^PA: physical activity.

^b^MET: metabolic equivalent of task.

^c^20/02/17-31/03/17.

^d^03/04/17-12/05/17.

^e^15/05/17-23/06/17.

^f^26/06/17-04/08/17.

^g^07/08/17-15/09/17.

^h^Exposure to intervention.

## Discussion

### Principal Findings

This study assessed the feasibility of implementing a primary care-based eHealth tool to screen for PA levels and provide tailored, evidence-based resources for both providers and patients. Over the course of 6 months, 530 patients were enrolled, with limited investment in personnel. Results show a trend toward improvement in PA levels for those who received the intervention, although the unexpectedly high variability limited statistical power. Few prior studies have successfully implemented a tool that both screens and provides tailored resources for PA in a primary care setting [24,62,63]. This study demonstrates the feasibility and potential of impact of using eHealth technology to deliver tailored, evidence-based care in primary care; a model that can be adapted to many health-promotion behaviors.

The high recruitment rate, including completion of emailed e-surveys, aligns with previous studies that suggest high acceptability among patients for using e-surveys to collect primary care data and integrate it into the EMR [64-66]. The process evaluation indicates that almost all patients in the intervention arm received counseling about PA, almost half received a PA prescription, and most of them were highly satisfied with the counseling they received. This suggests a potential for eHealth interventions to reduce barriers to screening, counseling, and self-management for health behaviors and to improve adherence to evidence-based treatment guidelines in a manner that is patient-centered [24,62,63].

However, intervention fidelity was not ideal: only a quarter of intervention patients received the customized toolkit with tailored messaging and resources from their PCP. These results suggest the existence of barriers to clinicians’ distribution of resource toolkits; this may include low perceived benefit of toolkit, poor intervention design, lack of education, or competing time pressures. Although all participating physicians received training before joining the intervention arm, it is possible that it was not sufficient, and further efforts to remind providers of the intervention would be required. It is possible that sending tailored information directly to patients before an appointment may facilitate shared decision-making on PA during the clinical encounter [67,68]. Exploring factors related to patient engagement and contextual factors impacting use in the clinic and patient context is also an important consideration that will be useful to evaluate in a larger trial [69].

The IPAQ-SF tool was selected to measure the primary outcome of this study, because of its frequent use and feasibility. The study attempted to control for previously documented concerns with high measurement variability with a large sample size [70]. However, PA levels as measured by the IPAQ-SF tool in our study exhibited higher than expected levels of variability in the data, making it difficult to attribute intervention effects. Although there is some evidence that accelerometers provide complementary or even superior PA tracking to self-reported tools [71], the limited resources of this pilot study precluded us from their use. Future studies of similar interventions may benefit from a composite outcome of PA levels to reduce variability and improve accuracy, including both self-reported measures and tracking using an accelerometer [72-74].

In addition to careful consideration of outcome measures, appropriate patient selection is an important consideration for future work in this area. It is possible that patients who attend clinic for a PHR may be systematically different (ie, biased toward interest or willingness to engage in healthy lifestyle behaviors) from the general population. PHR visits were used as they present a highly feasible time to incorporate structured counseling on PA. However, this potential bias may explain why patients in our study had much higher than expected levels of PA. It is also possible that focusing on these types of visits limits the potential for effectiveness of the intervention if PA is already routinely discussed during usual care. Unfortunately, resources were not available to capture process data from usual care patients in this pilot trial.

### Limitations

This pilot feasibility study has several important limitations including the low number of participating clusters resulting in few random assignments. Randomizing a small number of clusters can undermine the conventional benefits of randomization, resulting in increased risk of chance imbalances, increased type I and II error, and limited external validity [59]. Furthermore, within many cluster-periods, a limited number of participants were enrolled, which was reflected in the substantial observed variability in the primary outcome at the patient-level. In recognizing these limitations, we opted to use a cluster-level analysis to circumvent issues regarding patient- and PCP-level baseline imbalances and clustering of patient responses within teams. In aggregating to this level, however, the number of observations and corresponding statistical power was substantially reduced. As a SW-CRT design is suitable to test the effect of an intervention on PA, future studies must recruit a large number of clusters to minimize the aforementioned issues. This would enable using more conventional, mixed-effects regression that accounts for clustering via random effects and involves a greater number of observations (via avoiding aggregation) that can result in more power to detect treatment effects, if truly present [59], and adjust for baseline imbalances with reduced concern of overfitting. Furthermore, our process evaluation had a high level of missing data, particularly on the overall satisfaction question, increasing the risk of bias in the reported results. It is possible that those who were not satisfied with the intervention were less likely to respond. Steps to increase response rates to process measure surveys, including electronic delivery, should be considered for future work.

### Conclusions

Our pilot study demonstrates that it is feasible to build an eHealth tool that integrates both screening and tailored resource provision in primary care with good patient acceptability. Further work to better understand and address clinician barriers to resource distribution is needed. Future studies should include a greater number of clusters, improved methods for collecting process measures to reduce missing data, and more accurate measures for capturing PA levels.

## Appendix

Multimedia Appendix 1 Intervention description.

Multimedia Appendix 2 Training material.

Multimedia Appendix 3 Process evaluation.

Multimedia Appendix 4 Physical activity survey.

Multimedia Appendix 5 Process evaluation survey results.

Multimedia Appendix 6 Cross-sectional sample size per team and measurement period.

Multimedia Appendix 7 CONSORT-EHEALTH checklist (V 1.6.1).

## Abbreviations

eHealth: electronic health
EMR: electronic medical record
FPHC: Family Practice Health Centre
HAPA: health action process approach
IPAQ-SF: international physical activity questionnaire-short form
MD: mean difference
MET: metabolic equivalent of task
PA: physical activity
PCP: primary care provider
PHR: periodic health review
PR: mean proportion
SW-CRT: stepped wedge cluster randomized trial
