# Supplementary material for: Feasibility of an Electronic Health Tool to Promote Physical Activity in Primary Care: Pilot Cluster Randomized Controlled Trial
Source: J Med Internet Res. 2020 Feb 14;22(2):e15424. doi: 10.2196/15424 (PMC7055803; doi:10.2196/15424)
Supplement: Multimedia Appendix 1 [file jmir_v22i2e15424_app1.docx]

## Appendix 1: Intervention description with example prescription and toolkit

### TIDiER Checklist

| **No.** | **Item** |
| --- | --- |
| **1** | **BRIEF NAME** |
|  | Screening While You Wait: Physical Activity |
| **2** | **WHY** |
|  | Despite the evidence that physical inactivity is harmful, there remains inconsistent screening and counselling for physical activity (PA) in primary care. eHealth interventions may help overcome commonly reported barriers to screening and counselling for physical activity. |
|  | **WHAT** |
| **3** | ***Materials:*** Prior to the trial, primary care providers (PCPs) received paper-based training handouts including information about the intervention and a list of “frequently asked questions”. When a PCP’s patient participated in the intervention, they received an electronic medical record (EMR) based message alerting them to the new materials. Materials embedded in the EMR included: a summary of survey results, a patient handout with resources tailored to survey responses (in PDF form) and a customized exercise prescription. Participants received the toolkit and exercise prescription in paper form from their PCP. All handouts provided information about online and community-based resources that support achieving and maintaining recommended PA levels. |
| **4** | ***Procedures:*** Primary care provider participants were trained by a co-investigator on the research team. Training included how to interpret the survey summary in the ERM, and how to provide the exercise prescription and tailored handout to the patient. They were also given advice on how to discuss physical activity with their patient during the appointment. 2 weeks prior to their periodic health review (PHR) the patient received an email from their PCP (if an email address was present in the EMR) asking them to complete the baseline survey. If this was not completed by the day of the appointment a research assistant would approach them in the waiting room to complete the survey on a tablet. During the appointment, the provider could access the exercise prescription and tailored handout for patients in the EMR, and print these for the patient to take home. Providers could decide how and when to do this in the context of the full appointment. |
| **5** | **WHO PROVIDED** |
|  | Primary care providers administered the intervention to patients of their practice that had a scheduled PHR. Nurses, medical residents and clinic administrators also had access to the patient’s survey responses, prescription and toolkit via the EMR. |
| **6** | **HOW**  Electronic surveys were administered electronically via email, or in-person via tablet. These were generated using the OCEAN platform. The EMR survey results were automatically summarized in the patient’s chart using integration protocols developed by OCEAN. All intervention materials (handout, prescription) were automatically embedded in the EMR distributed in-person by the primary care provider to the patient. |
| **7** | **WHERE** |
|  | The intervention took place online and in clinic, before, during and after the appointment. The initial survey was distributed prior to the appointment via email, in the waiting room via tablet, or both (if there was no email response by the appointment date). Intervention materials were distributed in the appointment by the primary care provider. Patients randomized to the intervention group were given a paper-based process evaluation survey right after their appointment, or an online process evaluation survey via email 1-day after their appointment. All patients regardless of study-arm assignment, received a follow-up survey via email, mail, or phone 4-months after their appointment. |
| **8** | **WHEN and HOW MUCH** |
|  | The intervention was delivered once during the intervention patients’ PHR. 2 weeks after their PHR, they received the handout they were provided during their appointment via email in a pdf, or via mail in a letter. |
| **9** | **TAILORING** |
|  | Handouts were tailored based on survey responses. There were 5 possible handouts that were distributed based on the patient’s level of physical activity, perceived intention and self-efficacy to be physically active. Physical activity was measured using the self-reported physical activity level (IPAQ-SF) and compared to recommended PA levels of 150-minutes of moderate to vigorous exercise per week. Intention was measured using the question “I have made the decision to take part in a new kind of physical activity or increase my amount or intensity of physical activity soon.” Self-efficacy was measured using the average of the responses to 6 questions related by self-efficacy adapted from previous HAPA based behaviour change intervention. . For example, for relatively sedentary patients a ‘Getting Started’ toolkit was produced; for highly active patients the link was to a ‘Maintenance’ toolkit.  There were an additional 7 condition-specific toolkits that were sent in combination with the physical activity toolkit to address condition-specific physical activity considerations, such as mental health or respiratory conditions.  A tailored exercise prescription was also included in the EMR. If the patient reported less than 30 minutes of PA per week, the exercise prescription focused on starting activity including walking. Otherwise, the prescription enabled providers and patient to write down a target for moderate to vigorous activity. All prescriptions automatically included the patient’s self-reported reasons for wanting increase exercise. |
| **10** | **MODIFICATIONS** |
|  | N/A |
|  | **HOW WELL** |
| **11** | ***Planned:*** Process evaluations were distributed to intervention participants throughout the intervention. Participants were asked questions around the clinical encounter, if physical activity advice, resources and a prescription were delivered at their appointment, and by whom. |
| **12** | ***Actual:*** 61.8% of patients exposed to the intervention completed a process evaluation; of these patients, 49.4% reported receiving at least a prescription, 48.9% reported spending 2-5 minutes discussing PA with their provider, and 86.8% reported being satisfied with their PA discussion. |

### Example Prescription

### Example Toolkit
