# Supplementary figures and images for: Feasibility of an Electronic Health Tool to Promote Physical Activity in Primary Care: Pilot Cluster Randomized Controlled Trial
Source: J Med Internet Res. 2020 Feb 14;22(2):e15424. doi: 10.2196/15424 (PMC7055803; doi:10.2196/15424)

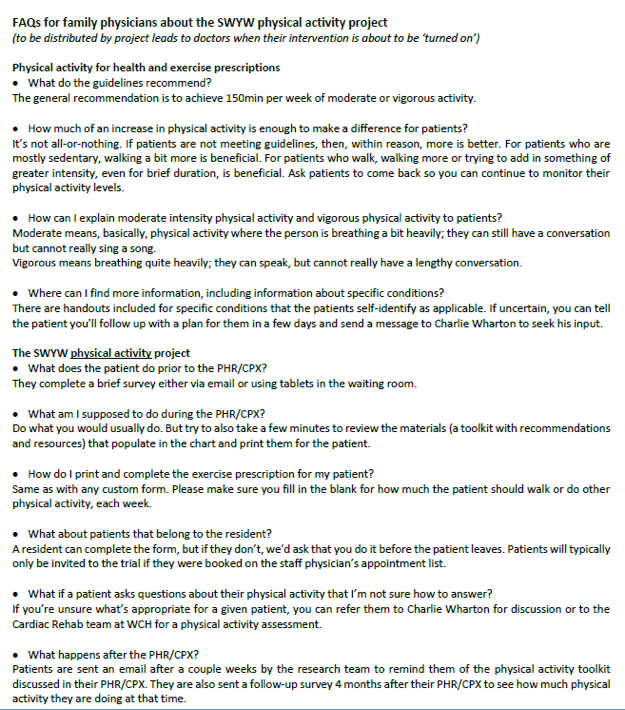

Supplement: Multimedia Appendix 2 [file jmir_v22i2e15424_app2.png]
