# Supplementary material for: Feasibility of an Electronic Health Tool to Promote Physical Activity in Primary Care: Pilot Cluster Randomized Controlled Trial
Source: J Med Internet Res. 2020 Feb 14;22(2):e15424. doi: 10.2196/15424 (PMC7055803; doi:10.2196/15424)
Supplement: Multimedia Appendix 3 [file jmir_v22i2e15424_app3.docx]

## Appendix 3: Process Evaluation

**Physical Activity Conversation Reminder**

Research shows that discussing exercise with your doctor can improve your health. Talk with your doctor about how your survey answers can be used create a plan or find options just for you. Ask for your personalized resources and recommendations about what, where, why and how to exercise!

1. **Did your doctor provide you with information about physical activity during your appointment? *(Please select one)***

🞏 Yes, I got an *Exercise Prescription* **and** some resources to take home

🞏 Yes, I got **only** an *Exercise Prescription* to take home

🞏 No, we discussed physical activity, but I wasn’t given anything to take home

🞏 No, we decided that it wasn’t really appropriate for me at this time

🞏 No, we didn’t really discuss physical activity

1. **How much time did your healthcare team spend focusing on physical activity during the appointment? *(Please select one)***

🞏 Not applicable, we did not discuss physical activity

🞏 less than 2 minutes

🞏 2-5 minutes

🞏 5-10 minutes

🞏 more than 10 minutes

**3. Who spoke with you about physical activity during your appointment? *(Please circle all that apply)***

| Staff Doctor | Trainee (resident or medical student) | Nurse | Not Applicable |
| --- | --- | --- | --- |

1. **Please rate your level of satisfaction with the physical activity conversation you had with your healthcare team *(Please circle one)***

| Very Satisfied | Somewhat Satisfied | Neutral | Somewhat Dissatisfied | Very Dissatisfied | Not Applicable |
| --- | --- | --- | --- | --- | --- |
