# Supplementary material for: Feasibility of an Electronic Health Tool to Promote Physical Activity in Primary Care: Pilot Cluster Randomized Controlled Trial
Source: J Med Internet Res. 2020 Feb 14;22(2):e15424. doi: 10.2196/15424 (PMC7055803; doi:10.2196/15424)
Supplement: Multimedia Appendix 4 [file jmir_v22i2e15424_app4.docx]

## Appendix 4: Physical Activity Survey

**Times conducted: Baseline, 4-month Follow-up**

**Physical Activity Survey**

The following survey is made up evidence-based questions to understand your current level of physical activity and your beliefs about physical activity. There are no right or wrong answers. The survey does not judge the amount of physical activity that is right for you. Please answer as openly and accurately as possible. The information will help us understand more about how family doctors can work with patients like you to make personalized recommendations about physical activity.

*Section 1*

Different people have different feelings about physical activity. We want to know what things you might find positive about physical activity and what things might get in the way of physical activity for you.

1. What are some things that make you want to be physically active?

Please select all of the statements below that apply to you:

*[tick box list]*

*--*

🞎 Physical activity is something I enjoy doing.

🞎 Physical activity decreases feeling of stress and tension for me.

🞎 Physical activity improves my mental health.

🞎 Physical activity improves my physical health.

🞎 Physical activity gives me a sense of personal accomplishment.

🞎 Physical activity lets me spend time with people I care about.

🞎 Exercising is a good way for me to meet new people.

🞎 Physical activity helps me manage my chronic condition.

🞎 Being physically active improves how I feel about myself.

🞎 Physical activity improves the quality of my work.

🞎 Other things motivate me to be physically active.

🞎 I am not motivated to be physically active at all.

1. What are some things that make it difficult to be physically active?

Please select all of the statements below that apply to you:

*[tick box list]*

*--*

🞎 I don’t have enough time to be physically active.

🞎 I get too tired by physical activity.

🞎 I don’t have access to places where I can be physically active.

🞎 I am too embarrassed to be physically active.

🞎 It costs too much to be physically active.

🞎 Places where I could be physically active do not have convenient schedules for me.

🞎 People I care about do not encourage me to be physically active.

🞎 My current pain, injury, or illness prevents me from be physically active.

🞎 I fear that I will fall or obtain an injury from being physically active.

🞎 Other things act as barriers to physical activity.

🞎 I have no barriers to physical activity at all.

[Agreed-upon statements regarding motivators and barriers flow into chart]

*Section 2*

For some people, certain medical conditions may make physical activity more important or more difficult. Have you ever been diagnosed with any of the following types of medical conditions?

*(Your doctor may already be aware of these conditions, but providing answers to this question helps us understand how we might personalize physical activity plans.)*

*(Select all that apply) [tick box list]*

--

🞎 Diabetes (e.g., Pre-Diabetes, Type 1, Type 2)

🞎 Cardiovascular condition (e.g., heart disease, high blood pressure, high cholesterol)

🞎 Respiratory condition (e.g., asthma, COPD)

🞎 Musculoskeletal condition (e.g., arthritis, lower back pain, fibromyalgia)

🞎 Mental health condition (e.g., depression, anxiety)

🞎 Neurological conditions (e.g., Multiple Sclerosis, Parkinson’s)

🞎 Cancer

🞎 None of the above

*Section 3*

We are interested in finding out about the kinds of physical activities that people do as part of their everyday lives. The questions will ask you about the time you spent being physically active in the **last 7 days.** If you have had a temporary illness (for the last week or two), think of the last week prior to your illness. Please answer each question even if you do not consider yourself to be an active person. Please think about the activities you do at work, as part of your house and yard work, to get from place to place, and in your spare time for recreation, physical activity or sport.

Think about all the **vigorous** activities that you did in the **last 7 days**. If you have had a temporary illness (for the last week or two), think of the last week prior to your illness. **Vigorous** physical activities refer to activities that take hard physical effort and make you breathe much harder than normal. Think *only* about those physical activities that you did for at least 20 minutes at a time.

1. During the **last 7 days**, on how many days did you do **vigorous** physical activities like heavy lifting, digging, aerobics, or fast bicycling?

*(Select one) [drop down list]*

--

0/No vigorous activity

1

2

3

4

5

6

7

1. How much time did you usually spend doing **vigorous** physical activities on one of those days?

*(Select one) [2 drop down lists; 1 for hours, 1 for minutes]*

--

[0-12] hours [0-59] minutes

Think about all the **moderate** activities that you did in the **last 7 days.** If you have had a temporary illness (for the last week or two), think of the last week prior to your illness. **Moderate** activities refer to activities that take moderate physical effort and make you breathe somewhat harder than normal. Think only about those physical activities that you did for at least 10 minutes at a time.

1. During the **last 7 days**, on how many days did you do **moderate** physical activities like carrying light loads, bicycling at a regular pace, or doubles tennis? Do not include walking.

*(Select one) [drop down list]*

--

0/No moderate activity

1

2

3

4

5

6

7

1. How much time did you usually spend doing *moderate* physical activities on one of those days?

*(Select one) [2 drop down lists; 1 for hours, 1 for minutes]*

--

[0-12] hours

[0-59] minutes

Think about the time you spend **walking** in the **last 7 days.** If you have had a temporary illness (for the last week or two), think of the last week prior to your illness. This includes at work and at home, walking to travel from place to place, and any other leisure walking that you have done solely for recreation, sport, physical activity, or leisure

1. During the **last 7 days,** on how many days did you **walk** for at least 10 minutes at a time?

*(Select one) [drop down list]*

--

0/No walking

1

2

3

4

5

6

7

1. How much time did you usually spend **walking** on one of those days?

*(Select one) [2 drop down lists; 1 for hours, 1 for minutes]*

--

[0-12] hours

[0-59] minutes

*Section 4*

*The following questions seek to understand how motivated you are to be physically active. Please remember, there are no right or wrong answers and the survey does not judge the amount of physical activity that is right for you. Please answer as openly and accurately as possible.*

1. I have made the decision to take part in a new kind of physical activity or increase my amount or intensity of physical activity soon.

*--*

[Yes/No] *[tick boxes]*

[If ‘Yes’ – Classify as “Intender”, go to *Question 2*, if ‘No’ classify as “Pre-intender”]

1. My health will be improved from more physical activity, even in small amounts.

*(Select one) [drop down list]*

*--*

Strongly disagree

Disagree

Agree

Strongly agree

*Section 5*

*The following questions seek to understand how confident you are about your ability to regularly be physically active.*

1. I am certain that I can be physically active on a regular basis, even when I don’t really feel like being physically active.

*(Select one) [drop down list]*

*--*

Strongly disagree

Disagree

Agree

Strongly agree

Not applicable, I generally feel like exercising

1. I am certain that I can be physically active on a regular basis, even if the physical activity is difficult.

*(Select one) [drop down list]*

*--*

Strongly disagree

Disagree

Agree

Strongly agree

Not applicable, physical activity is not difficult for me

1. I am capable of continuous physical activity on a regular basis even if I do not see or feel any positive changes.

*(Select one) [drop down list]*

*--*

Strongly disagree

Disagree

Agree

Strongly agree

Not applicable, I see and feel positive changes

1. I am capable of continuous physical activity on a regular basis even if people I care about are not physically active.

*(Select one) [drop down list]*

*--*

Strongly disagree

Disagree

Agree

Strongly agree

Not applicable, people I care about are very active

1. I am confident that I can return to a physically active lifestyle, even if I have been less physically active than I had planned on several occasions.

*(Select one) [drop down list]*

*--*

Strongly disagree

Disagree

Agree

Strongly agree

Not applicable, I do not have an active lifestyle

1. I am confident that I can return to a physically active lifestyle, even if I encounter obstacles that I did not anticipate.

*(Select one) [drop down list]*

*--*

Strongly disagree

Disagree

Agree

Strongly agree

Not applicable, I do not have an active lifestyle
