# Supplementary material for: Feasibility of an Electronic Health Tool to Promote Physical Activity in Primary Care: Pilot Cluster Randomized Controlled Trial
Source: J Med Internet Res. 2020 Feb 14;22(2):e15424. doi: 10.2196/15424 (PMC7055803; doi:10.2196/15424)
Supplement: Multimedia Appendix 5 [file jmir_v22i2e15424_app5.docx]

**Appendix 5: Process Evaluation Survey Results**

| **Table 2**. Responses to process evaluation survey questions by intervention patients per team. | | | | | |  |  |
| --- | --- | --- | --- | --- | --- | --- | --- |
|  | **Team** | | | |  | |  |
| **Process measure** | **1** | **2** | **3** | **4** | **Overall** | |  |
| 1. Provided take home PA materials, n (%) |  |  |  |  |  | |  |
| Yes, Rx + other resources | 6 (9.5) | 15 (24.2) | 15 (45.5) | 6 (30.0) | 42 (23.6) | |  |
| Yes, Rx only | 23 (36.5) | 19 (30.7) | 3 (9.1) | 1 (5.0) | 46 (25.8) | |  |
| No, currently not appropriate/needed | 4 (6.4) | 6 (9.7) | 1 (3.0) | 0 (0.0) | 11 (6.2) | |  |
| No, but discussed PA | 23 (36.5) | 20 (32.3) | 11 (33.3) | 13 (65.0) | 67 (37.6) | |  |
| No, no or minimal PA talk | 7 (11.1) | 2 (3.2) | 3 (9.1) | 0 (0.0) | 12 (6.7) | |  |
| Missing^b^ | 1 | 3 | 0 | 1 | 5 | |  |
| 2. Time spent on PA talk (minutes), n (%) |  |  |  |  |  | |  |
| <2 | 15 (23.8) | 20 (32.3) | 5 (16.7) | 10 (47.6) | 50 (28.4) | |  |
| 2-5 | 34 (54.0) | 31 (50.0) | 14 (46.7) | 7 (33.3) | 86 (48.9) | |  |
| >5 | 14 (22.2) | 11 (17.7) | 11 (36.6) | 4 (19.1) | 40 (22.7) | |  |
| N/A^b^ | 1 | 1 | 3 | 0 | 5 | |  |
| Missing^b^ | 0 | 2 | 0 | 0 | 2 | |  |
| 3. Health professional(s) involved in PA discussion: |  |  |  |  |  | |  |
| a. Staff physician, n (%) |  |  |  |  |  | |  |
| Yes | 44 (80.0) | 53 (82.8) | 29 (87.9) | 15 (75.0) | 141 (78.8) | |  |
| No | 18 (20.0) | 11 (17.2) | 4 (12.1) | 5 (25.0) | 38 (21.2) | |  |
| Missing^b^ | 2 | 1 | 0 | 1 | 4 | |  |
| b. Trainee, n (%) |  |  |  |  |  | |  |
| Yes | 10 (16.1) | 9 (14.1) | 3 (9.1) | 7 (35.0) | 29 (16.2) | |  |
| No | 52 (83.9) | 55 (85.9) | 30 (90.9) | 13 (65.0) | 150 (83.8) | |  |
| Missing^b^ | 2 | 1 | 0 | 1 | 4 | |  |
| c. Nurse, n (%) |  |  |  |  |  | |  |
| Yes | 40 (64.5) | 30 (46.9) | 13 (39.4) | 11 (55.0) | 94 (52.5) | |  |
| No | 22 (35.5) | 34 (53.1) | 20 (60.6) | 9 (45.0) | 85 (47.5) | |  |
| Missing^b^ | 2 | 1 | 0 | 1 | 4 | |  |
| 4. Satisfaction with PA talk, n (%) |  |  |  |  |  | |  |
| Dissatisfied^b^ | 0 | 0 | 0 | 0 | 0 | |  |
| Neutral | 3 (9.7) | 2 (6.45) | 0 (0.0) | 4 (21.1) | 9 (8.3) | |  |
| Satisfied | 28 (90.3) | 29 (93.6) | 27 (100.0) | 15 (78.9) | 99 (91.7) | |  |
| N/A^b^ | 1 | 1 | 3 | 1 | 6 | |  |
| Missing^b^ | 32 | 33 | 3 | 1 | 69 | |  |
| *Note*: * Significant at P-value≤0.05. Column percentages  ^a^ Compared proportion of any ‘Yes’ response to proportion of any ‘No’ response across teams.  ^b^ Observations excluded from contributing to column percentages, corresponding Chi-square or Fisher’s tests.  ^c^ Multiple options could be selected per patient. Therefore, each column has a maximum value of 183 (total # of patients completing process evaluation survey) - # missing. | | | | | |  |  |
