# Supplementary material for: Feasibility of an Electronic Health Tool to Promote Physical Activity in Primary Care: Pilot Cluster Randomized Controlled Trial
Source: J Med Internet Res. 2020 Feb 14;22(2):e15424. doi: 10.2196/15424 (PMC7055803; doi:10.2196/15424)
Supplement: Multimedia Appendix 6 [file jmir_v22i2e15424_app6.docx]

## Appendix 6: Cross-sectional sample size per team and measurement period.

|  | **No. of new participants per team (cluster) and period**  **(No. with follow-up data)** | | | | | |  | |  |
| --- | --- | --- | --- | --- | --- | --- | --- | --- | --- |
| **Team** | **Period 1**  **(20/02/17-31/03/17)** | | **Period 2**  **(03/04/17-12/05/17)** | **Period 3**  **(15/05/17-23/06/17)** | **Period 4**  **(26/06/17-04/08/17)** | **Period 5**  **(07/08/17-15/09/17)** | |  |  |
| 1 | 12 (9) | | 34 (29) | 28 (21) | 24 (20) | 33 (26) | |  |  |
| 2 | 18 (16) | | 26 (24) | 32 (25) | 41 (36) | 26 (21) | |  |  |
| 3 | 20 (16) | | 19 (16) | 31 (26) | 23 (15) | 25 (21) | |  |  |
| 4 | 18 (15) | | 24 (18) | 38 (33) | 28 (24) | 30 (26) | |  |  |
|  |  | |  |  |  |  | |  |  |
|  |  | Intervention condition | | | | | | | |
|  |  | Control condition | | | | | | | |
