# Supplementary material for: Feasibility of an Electronic Health Tool to Promote Physical Activity in Primary Care: Pilot Cluster Randomized Controlled Trial
Source: J Med Internet Res. 2020 Feb 14;22(2):e15424. doi: 10.2196/15424 (PMC7055803; doi:10.2196/15424)
Supplement: Multimedia Appendix 7 [file jmir_v22i2e15424_app7.pdf]

# CONSORT-EHEALTH (V 1.6.1) - Submission/Publication Form

The CONSORT-EHEALTH checklist is intended for authors of randomized trials evaluating web-based and Internet-based applications/interventions, including mobile interventions, electronic games (incl multiplayer games), social media, certain telehealth applications, and other interactive and/or networked electronic applications. Some of the items (e.g. all subitems under item 5 - description of the intervention) may also be applicable for other study designs.

The goal of the CONSORT EHEALTH checklist and guideline is to be
a) a guide for reporting for authors of RCTs,
b) to form a basis for appraisal of an ehealth trial (in terms of validity)

CONSORT-EHEALTH items/subitems are MANDATORY reporting items for studies published in the Journal of Medical Internet Research and other journals / scientific societies endorsing the checklist.

Items numbered 1., 2., 3., 4a., 4b etc are original CONSORT or CONSORT-NPT (non-pharmacologic treatment) items.
Items with Roman numerals (i., ii, iii, iv etc.) are CONSORT-EHEALTH extensions/clarifications.

As the CONSORT-EHEALTH checklist is still considered in a formative stage, we would ask that you also RATE ON A SCALE OF 1-5 how important/useful you feel each item is FOR THE PURPOSE OF THE CHECKLIST and reporting guideline (optional).

Mandatory reporting items are marked with a red *.
In the textboxes, either copy & paste the relevant sections from your manuscript into this form - please include any quotes from your manuscript in QUOTATION MARKS,
or answer directly by providing additional information not in the manuscript, or elaborating on why the item was not relevant for this study.

YOUR ANSWERS WILL BE PUBLISHED AS  A SUPPLEMENTARY FILE TO YOUR PUBLICATION IN JMIR AND ARE CONSIDERED PART OF YOUR PUBLICATION (IF ACCEPTED).
Please fill in these questions diligently. Information will not be copyedited, so please use proper spelling and grammar, use correct capitalization, and avoid abbreviations.

DO NOT FORGET TO SAVE AS PDF _AND_ CLICK THE SUBMIT BUTTON SO YOUR ANSWERS ARE IN OUR DATABASE !!!

Citation Suggestion (if you append the pdf as Appendix we suggest to cite this paper in the caption):
Eysenbach G, CONSORT-EHEALTH Group
CONSORT-EHEALTH: Improving and Standardizing Evaluation Reports of Web-based and Mobile Health Interventions
J Med Internet Res 2011;13(4):e126
URL: http://www.jmir.org/2011/4/e126/
doi: 10.2196/jmir.1923
PMID: 22209829

* Required

## Your name *

First Last

Payal Agarwal

## Primary Affiliation (short), City, Country *

University of Toronto, Toronto, Canada

Women's College Hospital, Toronto, Can

## Your e-mail address *

abc@gmail.com

payal.agarwal@wchospital.ca

## Title of your manuscript *

Provide the (draft) title of your manuscript.

Feasibility of an eHealth tool to promote physical activity in primary care: A cluster randomized controlled pilot trial

## Name of your App/Software/Intervention *

If there is a short and a long/alternate name, write the short name first and add the long name in brackets.

SWYW (Screening While You Wait)

## Evaluated Version (if any)

e.g. "V1", "Release 2017-03-01", "Version 2.0.27913"

Your answer

## Language(s) *

What language is the intervention/app in? If multiple languages are available, separate by comma (e.g. "English, French")

English

## URL of your Intervention Website or App

e.g. a direct link to the mobile app on app in appstore (itunes, Google Play), or URL of the website. If the intervention is a DVD or hardware, you can also link to an Amazon page.

Your answer

## URL of an image/screenshot (optional)

Your answer

## Accessibility *

Can an enduser access the intervention presently?

- ○ access is free and open
- ◉ access only for special usergroups, not open
- ○ access is open to everyone, but requires payment/subscription/in-app purchases
- ○ app/intervention no longer accessible
- ○ Other: ____________________

## Primary Medical Indication/Disease/Condition *

e.g. "Stress", "Diabetes", or define the target group in brackets after the condition, e.g. "Autism (Parents of children with)", "Alzheimers (Informal Caregivers of)"

Physical Activity in Primary Care

## Primary Outcomes measured in trial *

comma-separated list of primary outcomes reported in the trial

Physical Activity, measured by Metaboli

## Secondary/other outcomes

Are there any other outcomes the intervention is expected to affect?

Self-efficacy (action, recovery, maintenance and overall), Intention to exercise

## Recommended "Dose" *

What do the instructions for users say on how often the app should be used?

- ○ Approximately Daily
- ○ Approximately Weekly
- ○ Approximately Monthly
- ○ Approximately Yearly
- ◉ "as needed"
- ○ Other: ____

Approx. Percentage of Users (starters) still using the app as recommended after 3 months *

- (selected) unknown / not evaluated
- 0-10%
- 11-20%
- 21-30%
- 31-40%
- 41-50%
- 51-60%
- 61-70%
- 71%-80%
- 81-90%
- 91-100%
- Other: ____________________

Overall, was the app/intervention effective? *

- ( ) yes: all primary outcomes were significantly better in intervention group vs control
- ( ) partly: SOME primary outcomes were significantly better in intervention group vs control
- (•) no statistically significant difference between control and intervention
- ( ) potentially harmful: control was significantly better than intervention in one or more outcomes
- ( ) inconclusive: more research is needed
- ( ) Other: ________

Article Preparation Status/Stage *

At which stage in your article preparation are you currently (at the time you fill in this form)

- ( ) not submitted yet - in early draft status
- (•) not submitted yet - in late draft status, just before submission
- ( ) submitted to a journal but not reviewed yet
- ( ) submitted to a journal and after receiving initial reviewer comments
- ( ) submitted to a journal and accepted, but not published yet
- ( ) published
- ( ) Other: ________

## Journal *

If you already know where you will submit this paper (or if it is already submitted), please provide the journal name (if it is not JMIR, provide the journal name under "other")

- ○ not submitted yet / unclear where I will submit this
- ● Journal of Medical Internet Research (JMIR)
- ○ JMIR mHealth and UHealth
- ○ JMIR Serious Games
- ○ JMIR Mental Health
- ○ JMIR Public Health
- ○ JMIR Formative Research
- ○ Other JMIR sister journal
- ○ Other: ____________________

## Is this a full powered effectiveness trial or a pilot/feasibility trial? *

- ● Pilot/feasibility
- ○ Fully powered

## Manuscript tracking number *

If this is a JMIR submission, please provide the manuscript tracking number under "other" (The ms tracking number can be found in the submission acknowledgement email, or when you login as author in JMIR. If the paper is already published in JMIR, then the ms tracking number is the four-digit number at the end of the DOI, to be found at the bottom of each published article in JMIR)

- ● no ms number (yet) / not (yet) submitted to / published in JMIR
- ○ Other: ____________________

## TITLE AND ABSTRACT

## 1a) TITLE: Identification as a randomized trial in the title

### 1a) Does your paper address CONSORT item 1a? *

I.e does the title contain the phrase "Randomized Controlled Trial"? (if not, explain the reason under "other")

(●) yes

( ) Other:

### 1a-i) Identify the mode of delivery in the title

Identify the mode of delivery. Preferably use “web-based” and/or “mobile” and/or “electronic game” in the title. Avoid ambiguous terms like “online”, “virtual”, “interactive”. Use “Internet-based” only if Intervention includes non-web-based Internet components (e.g. email), use “computer-based” or “electronic” only if offline products are used. Use “virtual” only in the context of “virtual reality” (3-D worlds). Use “online” only in the context of “online support groups”. Complement or substitute product names with broader terms for the class of products (such as “mobile” or “smart phone” instead of “iphone”), especially if the application runs on different platforms.

| | 1 | 2 | 3 | 4 | 5 | |
|---|---|---|---|---|---|---|
| subitem not at all important | ( ) | ( ) | (●) | ( ) | ( ) | essential |

### Does your paper address subitem 1a-i? *

Copy and paste relevant sections from manuscript title (include quotes in quotation marks "like this" to indicate direct quotes from your manuscript), or elaborate on this item by providing additional information not in the ms, or briefly explain why the item is not applicable/relevant for your study

We have not included the mode of delivery in the title as the surveys were administered via email or tablet, and the tool was embedded in the electronic medical record (EMR). These modes of delivery could not be appropriately summarized in the title.

## 1a-ii) Non-web-based components or important co-interventions in title

Mention non-web-based components or important co-interventions in title, if any (e.g., “with telephone support”).

| | 1 | 2 | 3 | 4 | 5 | |
|---|---|---|---|---|---|---|
| subitem not at all important | ○ | ○ | ◉ | ○ | ○ | essential |

## Does your paper address subitem 1a-ii?

Copy and paste relevant sections from manuscript title (include quotes in quotation marks "like this" to indicate direct quotes from your manuscript), or elaborate on this item by providing additional information not in the ms, or briefly explain why the item is not applicable/relevant for your study

Non-web-based components were not included in the title (same rationale as above).

## 1a-iii) Primary condition or target group in the title

Mention primary condition or target group in the title, if any (e.g., “for children with Type I Diabetes”) Example: A Web-based and Mobile Intervention with Telephone Support for Children with Type I Diabetes: Randomized Controlled Trial

| | 1 | 2 | 3 | 4 | 5 | |
|---|---|---|---|---|---|---|
| subitem not at all important | ○ | ○ | ○ | ○ | ◉ | essential |

## Does your paper address subitem 1a-iii? *

Copy and paste relevant sections from manuscript title (include quotes in quotation marks "like this" to indicate direct quotes from your manuscript), or elaborate on this item by providing additional information not in the ms, or briefly explain why the item is not applicable/relevant for your study

"physical activity in primary care"

# 1b) ABSTRACT: Structured summary of trial design, methods, results, and conclusions

NPT extension: Description of experimental treatment, comparator, care providers, centers, and blinding status.

## 1b-i) Key features/functionalities/components of the intervention and comparator in the METHODS section of the ABSTRACT

Mention key features/functionalities/components of the intervention and comparator in the abstract. If possible, also mention theories and principles used for designing the site. Keep in mind the needs of systematic reviewers and indexers by including important synonyms. (Note: Only report in the abstract what the main paper is reporting. If this information is missing from the main body of text, consider adding it)

| | 1 | 2 | 3 | 4 | 5 | |
|---|---|---|---|---|---|---|
| subitem not at all important | ○ | ○ | ○ | ○ | ◉ | essential |

## Does your paper address subitem 1b-i? *

Copy and paste relevant sections from the manuscript abstract (include quotes in quotation marks "like this" to indicate direct quotes from your manuscript), or elaborate on this item by providing additional information not in the ms, or briefly explain why the item is not applicable/relevant for your study

"The eHealth tool involved an electronic survey sent to the patients prior to their PHR via email or tablet; data were used to automatically produce tailored resources and a PA prescription in the electronic medical record of participants assigned to the intervention arm. Participants assigned to the control arm received usual care. Intervention feasibility was assessed by the proportion of completed surveys and patient-reported acceptability and fidelity measures. The primary effectiveness outcome was patient-reported PA at four months post-PHR, measured as Metabolic Equivalent of Task (MET) minutes. Secondary outcomes assessed determinants of PA, including self-efficacy and intention to change based on Health Action Process Approach (HAPA) behaviour change theory."

## 1b-ii) Level of human involvement in the METHODS section of the ABSTRACT

Clarify the level of human involvement in the abstract, e.g., use phrases like “fully automated” vs. “therapist/nurse/care provider/physician-assisted” (mention number and expertise of providers involved, if any). (Note: Only report in the abstract what the main paper is reporting. If this information is missing from the main body of text, consider adding it)

| | 1 | 2 | 3 | 4 | 5 | |
|---|---|---|---|---|---|---|
| subitem not at all important | ○ | ○ | ○ | ○ | ◉ | essential |

## Does your paper address subitem 1b-ii?

Copy and paste relevant sections from the manuscript abstract (include quotes in quotation marks "like this" to indicate direct quotes from your manuscript), or elaborate on this item by providing additional information not in the ms, or briefly explain why the item is not applicable/relevant for your study

Electronic surveys automatically produced resources, which could then be accessed by the patient's primary care provider during their annual periodic health review (PHR): "The eHealth tool involved an electronic survey sent to the patients prior to their PHR via email or tablet; data were used to automatically produce tailored resources and a PA prescription in the electronic medical record of participants assigned to the intervention arm."

## 1b-iii) Open vs. closed, web-based (self-assessment) vs. face-to-face assessments in the METHODS section of the ABSTRACT

Mention how participants were recruited (online vs. offline), e.g., from an open access website or from a clinic or a closed online user group (closed usergroup trial), and clarify if this was a purely web-based trial, or there were face-to-face components (as part of the intervention or for assessment). Clearly say if outcomes were self-assessed through questionnaires (as common in web-based trials). Note: In traditional offline trials, an open trial (open-label trial) is a type of clinical trial in which both the researchers and participants know which treatment is being administered. To avoid confusion, use “blinded” or “unblinded” to indicated the level of blinding instead of “open”, as “open” in web-based trials usually refers to “open access” (i.e. participants can self-enrol). (Note: Only report in the abstract what the main paper is reporting. If this information is missing from the main body of text, consider adding it)

| | 1 | 2 | 3 | 4 | 5 | |
|---|---|---|---|---|---|---|
| subitem not at all important | ○ | ○ | ○ | ○ | ◉ | essential |

## Does your paper address subitem 1b-iii?

Copy and paste relevant sections from the manuscript abstract (include quotes in quotation marks "like this" to indicate direct quotes from your manuscript), or elaborate on this item by providing additional information not in the ms, or briefly explain why the item is not applicable/relevant for your study

"The eHealth tool involved an electronic survey sent to the patients prior to their PHR via email or tablet"

## 1b-iv) RESULTS section in abstract must contain use data

Report number of participants enrolled/assessed in each group, the use/uptake of the intervention (e.g., attrition/adherence metrics, use over time, number of logins etc.), in addition to primary/secondary outcomes. (Note: Only report in the abstract what the main paper is reporting. If this information is missing from the main body of text, consider adding it)

| | 1 | 2 | 3 | 4 | 5 | |
|---|---|---|---|---|---|---|
| subitem not at all important | ○ | ○ | ○ | ○ | ◉ | essential |

## Does your paper address subitem 1b-iv?

Copy and paste relevant sections from the manuscript abstract (include quotes in quotation marks "like this" to indicate direct quotes from your manuscript), or elaborate on this item by providing additional information not in the ms, or briefly explain why the item is not applicable/relevant for your study

"1028 patients belonging to 34 physicians were invited to participate and 530 (51.6%) consented (intervention n=296, control n=234)."

## 1b-v) CONCLUSIONS/DISCUSSION in abstract for negative trials

Conclusions/Discussions in abstract for negative trials: Discuss the primary outcome - if the trial is negative (primary outcome not changed), and the intervention was not used, discuss whether negative results are attributable to lack of uptake and discuss reasons. (Note: Only report in the abstract what the main paper is reporting. If this information is missing from the main body of text, consider adding it)

| | 1 | 2 | 3 | 4 | 5 | |
|---|---|---|---|---|---|---|
| subitem not at all important | ○ | ○ | ○ | ○ | ◉ | essential |

## Does your paper address subitem 1b-v?

Copy and paste relevant sections from the manuscript abstract (include quotes in quotation marks "like this" to indicate direct quotes from your manuscript), or elaborate on this item by providing additional information not in the ms, or briefly explain why the item is not applicable/relevant for your study

"Our results suggest that it is feasible to build an eHealth tool that screens and provides tailored resources for PA in a primary care setting but sub-optimal intervention fidelity suggests greater work must be done to address physician barriers to resource distribution. Participant responses to the primary effectiveness outcome (MET-minutes) were highly variable, reflecting a need for more robust measures of PA in future trials to address limitations in patient-reported data."

## INTRODUCTION

## 2a) In INTRODUCTION: Scientific background and explanation of rationale

### 2a-i) Problem and the type of system/solution

Describe the problem and the type of system/solution that is object of the study: intended as stand-alone intervention vs. incorporated in broader health care program? Intended for a particular patient population? Goals of the intervention, e.g., being more cost-effective to other interventions, replace or complement other solutions? (Note: Details about the intervention are provided in "Methods" under 5)

| | 1 | 2 | 3 | 4 | 5 | |
|---|---|---|---|---|---|---|
| subitem not at all important | ○ | ○ | ○ | ○ | ◉ | essential |

### Does your paper address subitem 2a-i? *

Copy and paste relevant sections from the manuscript (include quotes in quotation marks "like this" to indicate direct quotes from your manuscript), or elaborate on this item by providing additional information not in the ms, or briefly explain why the item is not applicable/relevant for your study

"Primary care physicians (PCPs) are ideally positioned to positively affect levels of PA among their patients [8]. Multiple clinical guidelines recommend PCPs screen patients for current activity levels and offer targeted counselling during routine visits [9-12]. Evidence indicates that a tailored PA prescription from PCPs can improve overall activity levels [13-15]. Unfortunately, this is rarely implemented in real-world clinical practice [16-19], with reported barriers including lack of time, knowledge, and training in PA counselling, and a perceived inability to change patient behavior [20,21]. "
"Electronic screening of health behaviours can save time for PCPs and has been highly accepted by patients as a method to share information with their care team [22-24]. Furthermore, using computers to deliver tailored messaging and resources to patients can have a positive impact on behaviour change, including PA, relative to more traditional methods of health counselling [25-30]. Integrating screening and tailored information provision into one intervention may help change PA levels by addressing the complex needs of both providers and patients [24,25]."

## 2a-ii) Scientific background, rationale: What is known about the (type of) system

Scientific background, rationale: What is known about the (type of) system that is the object of the study (be sure to discuss the use of similar systems for other conditions/diagnoses, if appropiate), motivation for the study, i.e. what are the reasons for and what is the context for this specific study, from which stakeholder viewpoint is the study performed, potential impact of findings [2]. Briefly justify the choice of the comparator.

| | 1 | 2 | 3 | 4 | 5 | |
|---|---|---|---|---|---|---|
| subitem not at all important | ○ | ○ | ○ | ○ | ◉ | essential |

### Does your paper address subitem 2a-ii? *

Copy and paste relevant sections from the manuscript (include quotes in quotation marks "like this" to indicate direct quotes from your manuscript), or elaborate on this item by providing additional information not in the ms, or briefly explain why the item is not applicable/relevant for your study

"Electronic screening of health behaviours can save time for PCPs and has been highly accepted by patients as a method to share information with their care team [22-24]. Furthermore, using computers to deliver tailored messaging and resources to patients can have a positive impact on behaviour change, including PA, relative to more traditional methods of health counselling [25-30]. Integrating screening and tailored information provision into one intervention may help change PA levels by addressing the complex needs of both providers and patients [24,25]."

## 2b) In INTRODUCTION: Specific objectives or hypotheses

### Does your paper address CONSORT subitem 2b? *

Copy and paste relevant sections from the manuscript (include quotes in quotation marks "like this" to indicate direct quotes from your manuscript), or elaborate on this item by providing additional information not in the ms, or briefly explain why the item is not applicable/relevant for your study

"Our aim was to optimize the intervention, evaluate recruitment and retention of participants, and assess suitability of the primary outcome for a subsequent, larger definitive trial [31,32]."

## METHODS

## 3a) Description of trial design (such as parallel, factorial) including allocation ratio

### Does your paper address CONSORT subitem 3a? *

Copy and paste relevant sections from the manuscript (include quotes in quotation marks "like this" to indicate direct quotes from your manuscript), or elaborate on this item by providing additional information not in the ms, or briefly explain why the item is not applicable/relevant for your study

Stepped Wedge Cluster Randomized Controlled Trial

## 3b) Important changes to methods after trial commencement (such as eligibility criteria), with reasons

### Does your paper address CONSORT subitem 3b? *

Copy and paste relevant sections from the manuscript (include quotes in quotation marks "like this" to indicate direct quotes from your manuscript), or elaborate on this item by providing additional information not in the ms, or briefly explain why the item is not applicable/relevant for your study

"We conducted a pilot study using a pragmatic SW-CRT design to identify potential issues with implementation or analysis that might challenge the feasibility of future trials involving more clusters [35]."

### 3b-i) Bug fixes, Downtimes, Content Changes

Bug fixes, Downtimes, Content Changes: ehealth systems are often dynamic systems. A description of changes to methods therefore also includes important changes made on the intervention or comparator during the trial (e.g., major bug fixes or changes in the functionality or content) (5-iii) and other "unexpected events" that may have influenced study design such as staff changes, system failures/downtimes, etc. [2].

| | 1 | 2 | 3 | 4 | 5 | |
|---|---|---|---|---|---|---|
| subitem not at all important | ○ | ○ | ◉ | ○ | ○ | essential |

### Does your paper address subitem 3b-i?

Copy and paste relevant sections from the manuscript (include quotes in quotation marks "like this" to indicate direct quotes from your manuscript), or elaborate on this item by providing additional information not in the ms, or briefly explain why the item is not applicable/relevant for your study

There were no bug fixes or downtimes to report during the study period.

## 4a) Eligibility criteria for participants

### Does your paper address CONSORT subitem 4a? *

Copy and paste relevant sections from the manuscript (include quotes in quotation marks "like this" to indicate direct quotes from your manuscript), or elaborate on this item by providing additional information not in the ms, or briefly explain why the item is not applicable/relevant for your study

Your answer

This is a required question

### 4a-i) Computer / Internet literacy

Computer / Internet literacy is often an implicit “de facto” eligibility criterion - this should be explicitly clarified.

| | 1 | 2 | 3 | 4 | 5 | |
|---|---|---|---|---|---|---|
| subitem not at all important | ○ | ○ | ○ | ○ | ◉ | essential |

### Does your paper address subitem 4a-i?

Copy and paste relevant sections from the manuscript (include quotes in quotation marks "like this" to indicate direct quotes from your manuscript), or elaborate on this item by providing additional information not in the ms, or briefly explain why the item is not applicable/relevant for your study

"Patients rostered to a participating physician were eligible if they attended a periodic health review (PHR) during the study period and were aged 18 to 79 at the time of the PHR. PHRs were considered appropriate opportunities to deliver the intervention, as they focus on preventative care counselling [39]. Patients deemed unable to safely or effectively complete the intervention at the time of their PHR were excluded. This included those with dementia or cognitive impairment, major active illness, and/or those who were pregnant. Non-English speakers were also excluded due to a lack of resources to appropriately accommodate other languages."

## 4a-ii) Open vs. closed, web-based vs. face-to-face assessments:

Open vs. closed, web-based vs. face-to-face assessments: Mention how participants were recruited (online vs. offline), e.g., from an open access website or from a clinic, and clarify if this was a purely web-based trial, or there were face-to-face components (as part of the intervention or for assessment), i.e., to what degree got the study team to know the participant. In online-only trials, clarify if participants were quasi-anonymous and whether having multiple identities was possible or whether technical or logistical measures (e.g., cookies, email confirmation, phone calls) were used to detect/prevent these.

| | 1 | 2 | 3 | 4 | 5 | |
|---|---|---|---|---|---|---|
| subitem not at all important | ○ | ○ | ○ | ○ | ◉ | essential |

## Does your paper address subitem 4a-ii? *

Copy and paste relevant sections from the manuscript (include quotes in quotation marks "like this" to indicate direct quotes from your manuscript), or elaborate on this item by providing additional information not in the ms, or briefly explain why the item is not applicable/relevant for your study

"All patients deemed eligible for the study received an email two weeks prior to their visit with a link to a secure electronic survey (e-survey). Those who did not complete the survey prior to their appointment were approached in clinic and the e-survey was completed using a digital tablet."

## 4a-iii) Information giving during recruitment

Information given during recruitment. Specify how participants were briefed for recruitment and in the informed consent procedures (e.g., publish the informed consent documentation as appendix, see also item X26), as this information may have an effect on user self-selection, user expectation and may also bias results.

| | 1 | 2 | 3 | 4 | 5 | |
|---|---|---|---|---|---|---|
| subitem not at all important | ○ | ○ | ◉ | ○ | ○ | essential |

## Does your paper address subitem 4a-iii?

Copy and paste relevant sections from the manuscript (include quotes in quotation marks "like this" to indicate direct quotes from your manuscript), or elaborate on this item by providing additional information not in the ms, or briefly explain why the item is not applicable/relevant for your study

"The e-survey collected informed consent, assessed baseline PA, and assessed perceived barriers and motivators for PA."

# 4b) Settings and locations where the data were collected

## Does your paper address CONSORT subitem 4b? *

Copy and paste relevant sections from the manuscript (include quotes in quotation marks "like this" to indicate direct quotes from your manuscript), or elaborate on this item by providing additional information not in the ms, or briefly explain why the item is not applicable/relevant for your study

Patients completed the survey in their own location (if by email) or in clinic on the day of their periodic health review.

## 4b-i) Report if outcomes were (self-)assessed through online questionnaires

Clearly report if outcomes were (self-)assessed through online questionnaires (as common in web-based trials) or otherwise.

| | 1 | 2 | 3 | 4 | 5 | |
|---|---|---|---|---|---|---|
| subitem not at all important | ○ | ○ | ○ | ○ | ● | essential |

## Does your paper address subitem 4b-i? *

Copy and paste relevant sections from the manuscript (include quotes in quotation marks "like this" to indicate direct quotes from your manuscript), or elaborate on this item by providing additional information not in the ms, or briefly explain why the item is not applicable/relevant for your study

"After exposure to the intervention, patients received a paper survey immediately after their appointment or e-survey one day after to collect process measures"
"The primary effectiveness outcome was patient-reported PA at four months post-PHR, measured as Metabolic Equivalent of Task (MET) minutes per week using the International Physical Activity Questionnaire – Short Form (IPAQ-SF) [48]. "
"An e-survey collected responses for both primary and secondary outcomes, and data was securely transferred and collated into a single, study-specific database (see Appendix 4 for survey)."

## 4b-ii) Report how institutional affiliations are displayed

Report how institutional affiliations are displayed to potential participants [on ehealth media], as affiliations with prestigious hospitals or universities may affect volunteer rates, use, and reactions with regards to an intervention.(Not a required item – describe only if this may bias results)

| | 1 | 2 | 3 | 4 | 5 | |
|---|---|---|---|---|---|---|
| subitem not at all important | ○ | ○ | ● | ○ | ○ | essential |

## Does your paper address subitem 4b-ii?

Copy and paste relevant sections from the manuscript (include quotes in quotation marks "like this" to indicate direct quotes from your manuscript), or elaborate on this item by providing additional information not in the ms, or briefly explain why the item is not applicable/relevant for your study

No institutional affiliations were displayed on intervention materials.

# 5) The interventions for each group with sufficient details to allow replication, including how and when they were actually administered

## 5-i) Mention names, credential, affiliations of the developers, sponsors, and owners

Mention names, credential, affiliations of the developers, sponsors, and owners [6] (if authors/evaluators are owners or developer of the software, this needs to be declared in a “Conflict of interest” section or mentioned elsewhere in the manuscript).

| | 1 | 2 | 3 | 4 | 5 | |
|---|---|---|---|---|---|---|
| subitem not at all important | ○ | ○ | ● | ○ | ○ | essential |

## Does your paper address subitem 5-i?

Copy and paste relevant sections from the manuscript (include quotes in quotation marks "like this" to indicate direct quotes from your manuscript), or elaborate on this item by providing additional information not in the ms, or briefly explain why the item is not applicable/relevant for your study

"We would like to thank ...Doug Kavanagh from Ocean by CognisantMD...for their assistance in developing the eHealth tool.

## 5-ii) Describe the history/development process

Describe the history/development process of the application and previous formative evaluations (e.g., focus groups, usability testing), as these will have an impact on adoption/use rates and help with interpreting results.

| | 1 | 2 | 3 | 4 | 5 | |
|---|---|---|---|---|---|---|
| subitem not at all important | ○ | ○ | ○ | ○ | ● | essential |

## Does your paper address subitem 5-ii?

Copy and paste relevant sections from the manuscript (include quotes in quotation marks "like this" to indicate direct quotes from your manuscript), or elaborate on this item by providing additional information not in the ms, or briefly explain why the item is not applicable/relevant for your study

"The intervention was refined using principles of user-centered design. This approach emphasizes the use of iterative product design with ongoing feedback from the end user to drive improvements and optimize acceptance and use of the tool [41-43]. This involved multiple interviews with potential end-users, as described in another paper [44]."

## 5-iii) Revisions and updating

Revisions and updating. Clearly mention the date and/or version number of the application/intervention (and comparator, if applicable) evaluated, or describe whether the intervention underwent major changes during the evaluation process, or whether the development and/or content was “frozen” during the trial. Describe dynamic components such as news feeds or changing content which may have an impact on the replicability of the intervention (for unexpected events see item 3b).

| | 1 | 2 | 3 | 4 | 5 | |
|---|---|---|---|---|---|---|
| subitem not at all important | ○ | ○ | ● | ○ | ○ | essential |

## Does your paper address subitem 5-iii?

Copy and paste relevant sections from the manuscript (include quotes in quotation marks "like this" to indicate direct quotes from your manuscript), or elaborate on this item by providing additional information not in the ms, or briefly explain why the item is not applicable/relevant for your study

There were no changes made to the intervention during the study period.

## 5-iv) Quality assurance methods

Provide information on quality assurance methods to ensure accuracy and quality of information provided [1], if applicable.

| | 1 | 2 | 3 | 4 | 5 | |
|---|---|---|---|---|---|---|
| subitem not at all important | ○ | ○ | ● | ○ | ○ | essential |

## Does your paper address subitem 5-iv?

Copy and paste relevant sections from the manuscript (include quotes in quotation marks "like this" to indicate direct quotes from your manuscript), or elaborate on this item by providing additional information not in the ms, or briefly explain why the item is not applicable/relevant for your study

We conducted a weekly quality assurance process where two research assistants would check the data from the database and compare it to the information populated in the intervention patient's chart. We also ensured that the participant was seen by the consenting PCP.

## 5-v) Ensure replicability by publishing the source code, and/or providing screenshots/screen-capture video, and/or providing flowcharts of the algorithms used

Ensure replicability by publishing the source code, and/or providing screenshots/screen-capture video, and/or providing flowcharts of the algorithms used. Replicability (i.e., other researchers should in principle be able to replicate the study) is a hallmark of scientific reporting.

| | 1 | 2 | 3 | 4 | 5 | |
|---|---|---|---|---|---|---|
| subitem not at all important | ○ | ○ | ● | ○ | ○ | essential |

## Does your paper address subitem 5-v?

Copy and paste relevant sections from the manuscript (include quotes in quotation marks "like this" to indicate direct quotes from your manuscript), or elaborate on this item by providing additional information not in the ms, or briefly explain why the item is not applicable/relevant for your study

We have not included the entire algorithm and toolkit possibilities as there are several. We are happy to provide these upon request.

## 5-vi) Digital preservation

Digital preservation: Provide the URL of the application, but as the intervention is likely to change or disappear over the course of the years; also make sure the intervention is archived (Internet Archive, webcitation.org, and/or publishing the source code or screenshots/videos alongside the article). As pages behind login screens cannot be archived, consider creating demo pages which are accessible without login.

| | 1 | 2 | 3 | 4 | 5 | |
|---|---|---|---|---|---|---|
| subitem not at all important | ○ | ○ | ● | ○ | ○ | essential |

## Does your paper address subitem 5-vi?

Copy and paste relevant sections from the manuscript (include quotes in quotation marks "like this" to indicate direct quotes from your manuscript), or elaborate on this item by providing additional information not in the ms, or briefly explain why the item is not applicable/relevant for your study

This tool is not publicly available. It is only available within the Women's College Hospital Family Practice Health Centre EMR, and will not disappear from this platform in the foreseeable future.

## 5-vii) Access

Access: Describe how participants accessed the application, in what setting/context, if they had to pay (or were paid) or not, whether they had to be a member of specific group. If known, describe how participants obtained "access to the platform and Internet" [1]. To ensure access for editors/reviewers/readers, consider to provide a "backdoor" login account or demo mode for reviewers/readers to explore the application (also important for archiving purposes, see vi).

| | 1 | 2 | 3 | 4 | 5 | |
|---|---|---|---|---|---|---|
| subitem not at all important | ○ | ○ | ◉ | ○ | ○ | essential |

## Does your paper address subitem 5-vii? *

Copy and paste relevant sections from the manuscript (include quotes in quotation marks "like this" to indicate direct quotes from your manuscript), or elaborate on this item by providing additional information not in the ms, or briefly explain why the item is not applicable/relevant for your study

Providers printed and handed the resources to intervention participants during their appointment.

## 5-viii) Mode of delivery, features/functionalities/components of the intervention and comparator, and the theoretical framework

Describe mode of delivery, features/functionalities/components of the intervention and comparator, and the theoretical framework [6] used to design them (instructional strategy [1], behaviour change techniques, persuasive features, etc., see e.g., [7, 8] for terminology). This includes an in-depth description of the content (including where it is coming from and who developed it) [1]," whether [and how] it is tailored to individual circumstances and allows users to track their progress and receive feedback" [6]. This also includes a description of communication delivery channels and – if computer-mediated communication is a component – whether communication was synchronous or asynchronous [6]. It also includes information on presentation strategies [1], including page design principles, average amount of text on pages, presence of hyperlinks to other resources, etc. [1].

| | 1 | 2 | 3 | 4 | 5 | |
|---|---|---|---|---|---|---|
| subitem not at all important | ○ | ○ | ○ | ◉ | ○ | essential |

## Does your paper address subitem 5-viii? *

Copy and paste relevant sections from the manuscript (include quotes in quotation marks "like this" to indicate direct quotes from your manuscript), or elaborate on this item by providing additional information not in the ms, or briefly explain why the item is not applicable/relevant for your study

"The intervention included three key components that were automatically generated based on the baseline survey. First, responses were summarized in the patient's EMR along with a statement comparing the results to current PA guidelines of 150 minutes of moderate to vigorous PA per week [12, 45, 46]. Second, the EMR was populated with a link to one of five toolkits which included online and community-based resources tailored to the patient's current PA levels and perceived barriers. Third, a customized PA prescription was generated based on current PA levels and patient identified motivators to increase PA. During the PHR, the prescription could be edited by the PCP based on discussions with the patient and then printed along with the toolkit for the patient to take home. Each patient's toolkit was also sent to them two weeks after the PHR via mail or email. A full description and examples of the prescription and toolkit can be found in Appendix 1 [47]."

## 5-ix) Describe use parameters

Describe use parameters (e.g., intended “doses” and optimal timing for use). Clarify what instructions or recommendations were given to the user, e.g., regarding timing, frequency, heaviness of use, if any, or was the intervention used ad libitum.

| | 1 | 2 | 3 | 4 | 5 | |
|---|---|---|---|---|---|---|
| subitem not at all important | ○ | ○ | ○ | ● | ○ | essential |

## Does your paper address subitem 5-ix?

Copy and paste relevant sections from the manuscript (include quotes in quotation marks "like this" to indicate direct quotes from your manuscript), or elaborate on this item by providing additional information not in the ms, or briefly explain why the item is not applicable/relevant for your study

"Second, the EMR was populated with a link to one of five toolkits which included online and community-based resources tailored to the patient’s current PA levels and perceived barriers. Third, a customized PA prescription was generated based on current PA levels and patient identified motivators to increase PA. During the PHR, the prescription could be edited by the PCP based on discussions with the patient and then printed along with the toolkit for the patient to take home. Each patient’s toolkit was also sent to them two weeks after the PHR via mail or email. A full description and examples of the prescription and toolkit can be found in Appendix 1 [47]."

## 5-x) Clarify the level of human involvement

Clarify the level of human involvement (care providers or health professionals, also technical assistance) in the e-intervention or as co-intervention (detail number and expertise of professionals involved, if any, as well as “type of assistance offered, the timing and frequency of the support, how it is initiated, and the medium by which the assistance is delivered”. It may be necessary to distinguish between the level of human involvement required for the trial, and the level of human involvement required for a routine application outside of a RCT setting (discuss under item 21 – generalizability).

| | 1 | 2 | 3 | 4 | 5 | |
|---|---|---|---|---|---|---|
| subitem not at all important | ○ | ○ | ○ | ○ | ● | essential |

## Does your paper address subitem 5-x?

Copy and paste relevant sections from the manuscript (include quotes in quotation marks "like this" to indicate direct quotes from your manuscript), or elaborate on this item by providing additional information not in the ms, or briefly explain why the item is not applicable/relevant for your study

"During the PHR, the prescription could be edited by the PCP based on discussions with the patient and then printed along with the toolkit for the patient to take home. Each patient's toolkit was also sent to them two weeks after the PHR via mail or email."

## 5-xi) Report any prompts/reminders used

Report any prompts/reminders used: Clarify if there were prompts (letters, emails, phone calls, SMS) to use the application, what triggered them, frequency etc. It may be necessary to distinguish between the level of prompts/reminders required for the trial, and the level of prompts/reminders for a routine application outside of a RCT setting (discuss under item 21 – generalizability).

| | 1 | 2 | 3 | 4 | 5 | |
|---|---|---|---|---|---|---|
| subitem not at all important | ○ | ○ | ● | ○ | ○ | essential |

## Does your paper address subitem 5-xi? *

Copy and paste relevant sections from the manuscript (include quotes in quotation marks "like this" to indicate direct quotes from your manuscript), or elaborate on this item by providing additional information not in the ms, or briefly explain why the item is not applicable/relevant for your study

Prompts and reminders were used to encourage participant survey completion. Email reminders and phone calls for a period of up to 6 weeks at 4 months post periodic health review (PHR), took place to decrease loss to follow-up.

## 5-xii) Describe any co-interventions (incl. training/support)

Describe any co-interventions (incl. training/support): Clearly state any interventions that are provided in addition to the targeted eHealth intervention, as ehealth intervention may not be designed as stand-alone intervention. This includes training sessions and support [1]. It may be necessary to distinguish between the level of training required for the trial, and the level of training for a routine application outside of a RCT setting (discuss under item 21 – generalizability.

| | 1 | 2 | 3 | 4 | 5 | |
|---|---|---|---|---|---|---|
| subitem not at all important | ○ | ○ | ○ | ○ | ● | essential |

Does your paper address subitem 5-xii? *

Copy and paste relevant sections from the manuscript (include quotes in quotation marks "like this" to indicate direct quotes from your manuscript), or elaborate on this item by providing additional information not in the ms, or briefly explain why the item is not applicable/relevant for your study

"To encourage intervention fidelity, one of the principal investigators (PA or NI) spoke with each of their PCP colleagues for 5 to 15 minutes prior to their cluster switching to the intervention arm. The intervention, including EMR outputs, were demonstrated using a 'test' patient chart in the EMR, and then a handout was reviewed that addressed both workflow integration and evidence for PA counselling (see Appendix 2 for handout)."

## 6a) Completely defined pre-specified primary and secondary outcome measures, including how and when they were assessed

## Does your paper address CONSORT subitem 6a? *

Copy and paste relevant sections from the manuscript (include quotes in quotation marks "like this" to indicate direct quotes from your manuscript), or elaborate on this item by providing additional information not in the ms, or briefly explain why the item is not applicable/relevant for your study

"After exposure to the intervention, patients received a paper survey immediately after their appointment or e-survey one day after to collect process measures (see Appendix 3). Acceptability was measured using a 5-point Likert scale ranging from 'very dissatisfied' to 'very satisfied'. Participants were also asked about the number of minutes of PA counselling they received (no discussion, less than 2 minutes, 2-5 minutes, 5-10 minutes, or more than 10 minutes), and if they received a PA prescription (yes/no). Feasibility was also assessed in part by number of eligible patients who completed a baseline survey and the frequency of missing or inaccurate data [35].

The primary effectiveness outcome was patient-reported PA at four months post-PHR, measured as Metabolic Equivalent of Task (MET) minutes per week using the International Physical Activity Questionnaire – Short Form (IPAQ-SF) [48]. The IPAQ-SF was selected for its short length, ease of administration, good test-retest reliability and low cost [48].

Secondary outcomes, also collected four months post-PHR, assessed attitudes towards PA using the HAPA constructs to guide assessment of proximal changes. Specifically, three sub-dimensions of self-efficacy (action, recovery, maintenance) were assessed, each measured via two questions (using a 4-point Likert scale ranging from 'strongly disagree' to 'strongly agree') [Roni to provide references]. A score for each sub-dimension of self-efficacy was calculated by summing the two questions, dividing by the maximum possible score and multiplying by 100 (for self-efficacy scores ranging from 0 to 100). The total self-efficacy score was the average of all sub-dimension scores.

Participants' intention regarding PA was measured in a two-step process. Those meeting recommended PA guidelines of 150 minutes of moderate to vigorous activity a week were defined as 'actors' [2]. Participants not meeting the recommended guidelines were defined as 'non-actors'. This group was further subdivided to 'intenders' and 'pre-intenders'. Those who agreed with the statement "I have made the decision to take part in a new kind of physical activity or increase my amount or intensity of physical activity soon" were deemed to be 'intenders', while those who disagreed were labelled 'pre-intenders'. An e-survey collected responses for both primary and secondary outcomes, and data was securely transferred and collated into a single, study-

## 6a-i) Online questionnaires: describe if they were validated for online use and apply CHERRIES items to describe how the questionnaires were designed/deployed

If outcomes were obtained through online questionnaires, describe if they were validated for online use and apply CHERRIES items to describe how the questionnaires were designed/deployed [9].

| | 1 | 2 | 3 | 4 | 5 | |
|---|---|---|---|---|---|---|
| subitem not at all important | ○ | ○ | ○ | ○ | ◉ | essential |

## Does your paper address subitem 6a-i?

Copy and paste relevant sections from manuscript text

"The primary effectiveness outcome was patient-reported PA at four months post-PHR, measured as Metabolic Equivalent of Task (MET) minutes per week using the International Physical Activity Questionnaire – Short Form (IPAQ-SF) [48]. The IPAQ-SF was selected for its short length, ease of administration, good test-retest reliability and low cost [48]."

## 6a-ii) Describe whether and how "use" (including intensity of use/dosage) was defined/measured/monitored

Describe whether and how "use" (including intensity of use/dosage) was defined/measured/monitored (logins, logfile analysis, etc.). Use/adoption metrics are important process outcomes that should be reported in any ehealth trial.

| | 1 | 2 | 3 | 4 | 5 | |
|---|---|---|---|---|---|---|
| subitem not at all important | ○ | ○ | ◉ | ○ | ○ | essential |

## Does your paper address subitem 6a-ii?

Copy and paste relevant sections from manuscript text

"After exposure to the intervention, patients received a paper survey immediately after their appointment or e-survey one day after to collect process measures (see Appendix 3). Acceptability was measured using a 5-point Likert scale ranging from 'very dissatisfied' to 'very satisfied'. Participants were also asked about the number of minutes of PA counselling they received (no discussion, less than 2 minutes, 2-5 minutes, 5-10 minutes, or more than 10 minutes), and if they received a PA prescription (yes/no). Feasibility was also assessed in part by number of eligible patients who completed a baseline survey and the frequency of missing or inaccurate data [35]."

## 6a-iii) Describe whether, how, and when qualitative feedback from participants was obtained

Describe whether, how, and when qualitative feedback from participants was obtained (e.g., through emails, feedback forms, interviews, focus groups).

| | 1 | 2 | 3 | 4 | 5 | |
|---|---|---|---|---|---|---|
| subitem not at all important | ○ | ○ | ○ | ○ | ◉ | essential |

## Does your paper address subitem 6a-iii?

Copy and paste relevant sections from manuscript text

"After exposure to the intervention, patients received a paper survey immediately after their appointment or e-survey one day after to collect process measures (see Appendix 3). Acceptability was measured using a 5-point Likert scale ranging from 'very dissatisfied' to 'very satisfied'. Participants were also asked about the number of minutes of PA counselling they received (no discussion, less than 2 minutes, 2-5 minutes, 5-10 minutes, or more than 10 minutes), and if they received a PA prescription (yes/no). Intervention feasibility was also assessed in part by number of eligible patients who completed a baseline survey and the frequency of missing or inaccurate data [35]."

# 6b) Any changes to trial outcomes after the trial commenced, with reasons

## Does your paper address CONSORT subitem 6b? *

Copy and paste relevant sections from the manuscript (include quotes in quotation marks "like this" to indicate direct quotes from your manuscript), or elaborate on this item by providing additional information not in the ms, or briefly explain why the item is not applicable/relevant for your study

N/A

# 7a) How sample size was determined

NPT: When applicable, details of whether and how the clustering by care provides or centers was addressed

## 7a-i) Describe whether and how expected attrition was taken into account when calculating the sample size

Describe whether and how expected attrition was taken into account when calculating the sample size.

| | 1 | 2 | 3 | 4 | 5 | |
|---|---|---|---|---|---|---|
| subitem not at all important | ○ | ○ | ○ | ○ | ○ | essential |

## Does your paper address subitem 7a-i?

Copy and paste relevant sections from manuscript title (include quotes in quotation marks "like this" to indicate direct quotes from your manuscript), or elaborate on this item by providing additional information not in the ms, or briefly explain why the item is not applicable/relevant for your study

Your answer

# 7b) When applicable, explanation of any interim analyses and stopping guidelines

## Does your paper address CONSORT subitem 7b? *

Copy and paste relevant sections from the manuscript (include quotes in quotation marks "like this" to indicate direct quotes from your manuscript), or elaborate on this item by providing additional information not in the ms, or briefly explain why the item is not applicable/relevant for your study

N/A

## 8a) Method used to generate the random allocation sequence

NPT: When applicable, how care providers were allocated to each trial group

### Does your paper address CONSORT subitem 8a? *

Copy and paste relevant sections from the manuscript (include quotes in quotation marks "like this" to indicate direct quotes from your manuscript), or elaborate on this item by providing additional information not in the ms, or briefly explain why the item is not applicable/relevant for your study

"Randomization occurred using computer-generated random numbers produced by an independent statistician [38]."

## 8b) Type of randomisation; details of any restriction (such as blocking and block size)

### Does your paper address CONSORT subitem 8b? *

Copy and paste relevant sections from the manuscript (include quotes in quotation marks "like this" to indicate direct quotes from your manuscript), or elaborate on this item by providing additional information not in the ms, or briefly explain why the item is not applicable/relevant for your study

"PCP teams functioning as naturally occurring clusters of clinicians and patients were randomized to allow gradual implementation of the tool and prevent intervention contamination across clusters [36-38]. The study was divided into five periods, each six weeks in length. Initially, no teams were exposed to the intervention [37], then one team was randomly assigned to begin the intervention at the start of each period [36]."

## 9) Mechanism used to implement the random allocation sequence (such as sequentially numbered containers), describing any steps taken to conceal the sequence until interventions were assigned

### Does your paper address CONSORT subitem 9? *

Copy and paste relevant sections from the manuscript (include quotes in quotation marks "like this" to indicate direct quotes from your manuscript), or elaborate on this item by providing additional information not in the ms, or briefly explain why the item is not applicable/relevant for your study

""Participants and researchers could not be blinded due to the nature of the intervention."

## 10) Who generated the random allocation sequence, who enrolled participants, and who assigned participants to interventions

### Does your paper address CONSORT subitem 10? *

Copy and paste relevant sections from the manuscript (include quotes in quotation marks "like this" to indicate direct quotes from your manuscript), or elaborate on this item by providing additional information not in the ms, or briefly explain why the item is not applicable/relevant for your study

"an independent statistician" produced the random allocation sequence, and research assistants enrolled participants in the intervention and control arms.

## 11a) If done, who was blinded after assignment to interventions (for example, participants, care providers, those assessing outcomes) and how

NPT: Whether or not administering co-interventions were blinded to group assignment

### 11a-i) Specify who was blinded, and who wasn’t

Specify who was blinded, and who wasn’t. Usually, in web-based trials it is not possible to blind the participants [1, 3] (this should be clearly acknowledged), but it may be possible to blind outcome assessors, those doing data analysis or those administering co-interventions (if any).

| | 1 | 2 | 3 | 4 | 5 | |
|---|---|---|---|---|---|---|
| subitem not at all important | ○ | ○ | ◉ | ○ | ○ | essential |

## Does your paper address subitem 11a-i? *

Copy and paste relevant sections from the manuscript (include quotes in quotation marks "like this" to indicate direct quotes from your manuscript), or elaborate on this item by providing additional information not in the ms, or briefly explain why the item is not applicable/relevant for your study

"Participants and researchers could not be blinded due to the nature of the intervention."

## 11a-ii) Discuss e.g., whether participants knew which intervention was the “intervention of interest” and which one was the “comparator”

Informed consent procedures (4a-ii) can create biases and certain expectations - discuss e.g., whether participants knew which intervention was the “intervention of interest” and which one was the “comparator”.

| | 1 | 2 | 3 | 4 | 5 | |
|---|---|---|---|---|---|---|
| subitem not at all important | ○ | ○ | ○ | ○ | ● | essential |

## Does your paper address subitem 11a-ii?

Copy and paste relevant sections from the manuscript (include quotes in quotation marks "like this" to indicate direct quotes from your manuscript), or elaborate on this item by providing additional information not in the ms, or briefly explain why the item is not applicable/relevant for your study

"Participants in the usual care group completed the same baseline questionnaire as the intervention group, but no EMR outputs or patient toolkits were produced. Participating PCPs were encouraged to provide PA advice (or not) as per their normal routines, i.e. no attempt was made to standardize usual care. PCPs received education about the intervention only in the week prior to the intervention being activated for their team." As this was described in the informed consent form, a participant would have been able to deduce whether or not they were in the intervention arm, based on the visit with their PCP.

# 11b) If relevant, description of the similarity of interventions

(this item is usually not relevant for ehealth trials as it refers to similarity of a placebo or sham intervention to a active medication/intervention)

## Does your paper address CONSORT subitem 11b? *

Copy and paste relevant sections from the manuscript (include quotes in quotation marks "like this" to indicate direct quotes from your manuscript), or elaborate on this item by providing additional information not in the ms, or briefly explain why the item is not applicable/relevant for your study

N/A

## 12a) Statistical methods used to compare groups for primary and secondary outcomes

NPT: When applicable, details of whether and how the clustering by care providers or centers was addressed

## Does your paper address CONSORT subitem 12a? *

Copy and paste relevant sections from the manuscript (include quotes in quotation marks "like this" to indicate direct quotes from your manuscript), or elaborate on this item by providing additional information not in the ms, or briefly explain why the item is not applicable/relevant for your study

"Analysis of the pilot data was mainly descriptive [35]. The distribution of patient- and physician-level baseline characteristics were summarized by team via means and standard deviations (median and interquartile range when skewed) and frequencies and proportions, respectively [37,38]."
"The presence of few clusters in our study limits options for estimating the preliminary effectiveness of the intervention. Specifically, it precludes the use of conventional analytic approaches for stepped wedge trials [33, 36, 37] that model patient-level responses while accounting for clustering via random effects, which require observations on many clusters to yield unbiased estimates and accurate standard errors [49,50]. Correspondingly, patient-level responses to the primary outcome to the cluster-period level were aggregated, which removes the need to adjust for patient- or physician-level characteristics or clustering of patient responses within teams [51]. To obtain a preliminary estimate of intervention effectiveness on the primary outcome, the cluster-period mean response was then regressed as the outcome using linear regression with intervention exposure as the primary independent variable and the following fixed effects included for as covariates: team (cluster); period; and mean baseline (or pretest) value [51]. To assess the robustness of our findings to statistical outliers, a sensitivity analysis was conducted involving regression analysis as specified for the primary outcome; however, prior to aggregating patient-level responses to the cluster-period level, those patient responses in the top 5% by primary outcome value were excluded.

Secondary outcomes were analyzed similarly. Preliminary treatment effect estimates on each self-efficacy measure (action, recovery, maintenance, and overall) at the cluster-period level via multivariable linear regression with adjustment for team, period, and baseline response as covariates were obtained. With respect to intention to change PA levels, the proportion of participants meeting criteria as an ‘actor’ or ‘intender’ at follow-up per cluster-period were calculated and expressed as a percentage. This value was then regressed as the outcome in a negative binomial regression model with intervention exposure as the primary independent variable, adjusting for period, team, and for the proportion meeting the outcome at baseline. A similar model focused only on the proportion of participants meeting criteria as an ‘actor’.

For each primary and secondary outcome, analysis was limited to patients who were randomized, attended their PHR, and provided baseline and follow-up data for that outcome. Statistical significance was assessed, where applicable, using a two-sided P-value of 0.05. SAS 9.4 (SAS Institute) was

## 12a-i) Imputation techniques to deal with attrition / missing values

Imputation techniques to deal with attrition / missing values: Not all participants will use the intervention/comparator as intended and attrition is typically high in ehealth trials. Specify how participants who did not use the application or dropped out from the trial were treated in the statistical analysis (a complete case analysis is strongly discouraged, and simple imputation techniques such as LOCF may also be problematic [4]).

| | 1 | 2 | 3 | 4 | 5 | |
|---|---|---|---|---|---|---|
| subitem not at all important | ○ | ○ | ○ | ○ | ◉ | essential |

## Does your paper address subitem 12a-i? *

Copy and paste relevant sections from the manuscript (include quotes in quotation marks "like this" to indicate direct quotes from your manuscript), or elaborate on this item by providing additional information not in the ms, or briefly explain why the item is not applicable/relevant for your study

"To assess the robustness of our findings to statistical outliers, a sensitivity analysis was conducted involving regression analysis as specified for the primary outcome; however, prior to aggregating patient-level responses to the cluster-period level, those patient responses in the top 5% by primary outcome value were excluded."

## 12b) Methods for additional analyses, such as subgroup analyses and adjusted analyses

## Does your paper address CONSORT subitem 12b? *

Copy and paste relevant sections from the manuscript (include quotes in quotation marks "like this" to indicate direct quotes from your manuscript), or elaborate on this item by providing additional information not in the ms, or briefly explain why the item is not applicable/relevant for your study

"The presence of few clusters in our study limits options for estimating the preliminary effectiveness of the intervention. Specifically, it precludes the use of conventional analytic approaches for stepped wedge trials [33, 36, 37] that model patient-level responses while accounting for clustering via random effects, which require observations on many clusters to yield unbiased estimates and accurate standard errors [49,50]. Correspondingly, patient-level responses to the primary outcome to the cluster-period level were aggregated, which removes the need to adjust for patient- or physician-level characteristics or clustering of patient responses within teams [51]."

# X26) REB/IRB Approval and Ethical Considerations [recommended as subheading under "Methods"] (not a CONSORT item)

## X26-i) Comment on ethics committee approval

| | 1 | 2 | 3 | 4 | 5 | |
|---|---|---|---|---|---|---|
| subitem not at all important | ○ | ○ | ○ | ○ | ◉ | essential |

## Does your paper address subitem X26-i?

Copy and paste relevant sections from the manuscript (include quotes in quotation marks "like this" to indicate direct quotes from your manuscript), or elaborate on this item by providing additional information not in the ms, or briefly explain why the item is not applicable/relevant for your study

"Research ethics approval was obtained from the Women's College Hospital Research Ethics Board (registered on ClinicalTrials.gov as NCT03181295)."

## x26-ii) Outline informed consent procedures

Outline informed consent procedures e.g., if consent was obtained offline or online (how? Checkbox, etc.?), and what information was provided (see 4a-ii). See [6] for some items to be included in informed consent documents.

| | 1 | 2 | 3 | 4 | 5 | |
|---|---|---|---|---|---|---|
| subitem not at all important | ○ | ○ | ○ | ○ | ◉ | essential |

## Does your paper address subitem X26-ii?

Copy and paste relevant sections from the manuscript (include quotes in quotation marks "like this" to indicate direct quotes from your manuscript), or elaborate on this item by providing additional information not in the ms, or briefly explain why the item is not applicable/relevant for your study

"The e-survey collected informed consent, assessed baseline PA, and assessed perceived barriers and motivators for PA." Informed consent was the first section of the electronic survey. If participants did not read and confirm their consent to the study, the survey automatically concluded.

## X26-iii) Safety and security procedures

Safety and security procedures, incl. privacy considerations, and any steps taken to reduce the likelihood or detection of harm (e.g., education and training, availability of a hotline)

| | 1 | 2 | 3 | 4 | 5 | |
|---|---|---|---|---|---|---|
| subitem not at all important | ○ | ○ | ○ | ○ | ◉ | essential |

## Does your paper address subitem X26-iii?

Copy and paste relevant sections from the manuscript (include quotes in quotation marks "like this" to indicate direct quotes from your manuscript), or elaborate on this item by providing additional information not in the ms, or briefly explain why the item is not applicable/relevant for your study

Community resources were a key component of the toolkits, and PCPs were encouraged to offer these resources to their patients. We also developed additional documents for those with comorbidities such as cardiovascular disease or cancer, to account for the unique challenges posed by other conditions on the ability to be physically active.
"Second, the EMR was populated with a link to one of five toolkits which included online and community-based resources tailored to the patient's current PA levels and perceived barriers, as well as an additional condition-specific PA toolkit if the patient reported any other condition (e.g. cardiovascular disease)."

# RESULTS

## 13a) For each group, the numbers of participants who were randomly assigned, received intended treatment, and were analysed for the primary outcome

NPT: The number of care providers or centers performing the intervention in each group and the number of patients treated by each care provider in each center

## Does your paper address CONSORT subitem 13a? *

Copy and paste relevant sections from the manuscript (include quotes in quotation marks "like this" to indicate direct quotes from your manuscript), or elaborate on this item by providing additional information not in the ms, or briefly explain why the item is not applicable/relevant for your study

"In total, 34 out of 39 eligible PCPs participated across the four teams in the clinic. Of 1,277 eligible patients, 1,028 were invited to participate and 948 consented. Randomization proceeded based on cluster allocation (see Figure 1). In total, 296/640 (46.3%) and 234/388 (60.3%) individuals randomized to the intervention and control groups respectively, completed the baseline survey and received their allocated treatment. Most participants (307/530 or 57.9%) completed the baseline survey via email prior to their PHR; 80/530 (15.1%) participants completed the survey via tablet (due to no email address in their EMR), and 143/530 (27.0%) completed the survey via tablet (after being sent the survey via email)."

# 13b) For each group, losses and exclusions after randomisation, together with reasons

## Does your paper address CONSORT subitem 13b? (NOTE: Preferably, this is shown in a CONSORT flow diagram) *

Copy and paste relevant sections from the manuscript (include quotes in quotation marks "like this" to indicate direct quotes from your manuscript), or elaborate on this item by providing additional information not in the ms, or briefly explain why the item is not applicable/relevant for your study

This is addressed in Figure 1: CONSORT flow diagram.

## 13b-i) Attrition diagram

Strongly recommended: An attrition diagram (e.g., proportion of participants still logging in or using the intervention/comparator in each group plotted over time, similar to a survival curve) or other figures or tables demonstrating usage/dose/engagement.

| | 1 | 2 | 3 | 4 | 5 | |
|---|---|---|---|---|---|---|
| subitem not at all important | ○ | ○ | ○ | ○ | ● | essential |

## Does your paper address subitem 13b-i?

Copy and paste relevant sections from the manuscript or cite the figure number if applicable (include quotes in quotation marks "like this" to indicate direct quotes from your manuscript), or elaborate on this item by providing additional information not in the ms, or briefly explain why the item is not applicable/relevant for your study

This can be found in Figure 1: CONSORT flow diagram.

# 14a) Dates defining the periods of recruitment and follow-up

## Does your paper address CONSORT subitem 14a? *

Copy and paste relevant sections from the manuscript (include quotes in quotation marks "like this" to indicate direct quotes from your manuscript), or elaborate on this item by providing additional information not in the ms, or briefly explain why the item is not applicable/relevant for your study

The recruitment periods can be found in figure 2. Follow-up occurred up to 6 weeks after the four month follow-up date.

## 14a-i) Indicate if critical "secular events" fell into the study period

Indicate if critical "secular events" fell into the study period, e.g., significant changes in Internet resources available or "changes in computer hardware or Internet delivery resources"

| | 1 | 2 | 3 | 4 | 5 | |
|---|---|---|---|---|---|---|
| subitem not at all important | ○ | ○ | ○ | ○ | ◉ | essential |

## Does your paper address subitem 14a-i?

Copy and paste relevant sections from the manuscript (include quotes in quotation marks "like this" to indicate direct quotes from your manuscript), or elaborate on this item by providing additional information not in the ms, or briefly explain why the item is not applicable/relevant for your study

No critical events occurred during the study period.

# 14b) Why the trial ended or was stopped (early)

### Does your paper address CONSORT subitem 14b? *

Copy and paste relevant sections from the manuscript (include quotes in quotation marks "like this" to indicate direct quotes from your manuscript), or elaborate on this item by providing additional information not in the ms, or briefly explain why the item is not applicable/relevant for your study

N/A

## 15) A table showing baseline demographic and clinical characteristics for each group

NPT: When applicable, a description of care providers (case volume, qualification, expertise, etc.) and centers (volume) in each group

### Does your paper address CONSORT subitem 15? *

Copy and paste relevant sections from the manuscript (include quotes in quotation marks "like this" to indicate direct quotes from your manuscript), or elaborate on this item by providing additional information not in the ms, or briefly explain why the item is not applicable/relevant for your study

Participant-level characteristics can be found in Table 1.

### 15-i) Report demographics associated with digital divide issues

In ehealth trials it is particularly important to report demographics associated with digital divide issues, such as age, education, gender, social-economic status, computer/Internet/ehealth literacy of the participants, if known.

| | 1 | 2 | 3 | 4 | 5 | |
|---|---|---|---|---|---|---|
| subitem not at all important | ○ | ○ | ○ | ○ | ◉ | essential |

### Does your paper address subitem 15-i? *

Copy and paste relevant sections from the manuscript (include quotes in quotation marks "like this" to indicate direct quotes from your manuscript), or elaborate on this item by providing additional information not in the ms, or briefly explain why the item is not applicable/relevant for your study

We reported the gender of our participants in Table 1.

## 16) For each group, number of participants (denominator) included in each analysis and whether the analysis was by original assigned groups

## 16-i) Report multiple “denominators” and provide definitions

Report multiple “denominators” and provide definitions: Report N’s (and effect sizes) “across a range of study participation [and use] thresholds” [1], e.g., N exposed, N consented, N used more than x times, N used more than y weeks, N participants “used” the intervention/comparator at specific pre-defined time points of interest (in absolute and relative numbers per group). Always clearly define “use” of the intervention.

| | 1 | 2 | 3 | 4 | 5 | |
|---|---|---|---|---|---|---|
| subitem not at all important | ○ | ○ | ○ | ○ | ◉ | essential |

## Does your paper address subitem 16-i? *

Copy and paste relevant sections from the manuscript (include quotes in quotation marks "like this" to indicate direct quotes from your manuscript), or elaborate on this item by providing additional information not in the ms, or briefly explain why the item is not applicable/relevant for your study

"In total, 183/296 (61.8%) of patients exposed to the intervention handed in a process evaluation survey following their PHR. Only 112 completed the survey fully, which represents 61.2% of those who handed in a survey and only 37.8% of the intervention group. Overall, fewer than half of respondents (88/178 or 49.4%) stated they received at least a PA prescription from their PCP. A chi-square test of independence indicated no significant difference in the proportion of patients who received at least a PA prescription versus no materials between teams ($\chi2(3)=3.03$; P-value=0.39). Among the 88 patients who received a PA prescription, just under half (42/88 or 47.7%) also received tailored resources to take home. The proportion of intervention patients who completed the process evaluation receiving both a PA prescription and resources ranged from a low of 9.4% (6/64 patients) for team 1 to a high of 45.5% (15/33 patients) for team 3.

Only 12/183 (6.6%) patients completing a process evaluation indicated that no PA discussion occurred during their appointment. Nearly half (86/176 or 48.9%) of participants who estimated the length of their PA discussion reported a length of 2-5 minutes, with patients in team 4 were more likely to report a talk of <2 minutes. Most patients reported being satisfied with their PA discussion irrespective of team, with no patients indicating they were dissatisfied. Of the process evaluation questions, patient satisfaction with their PA counselling (if applicable) was most prone to missing responses, with only 114/183 (62.3%) providing a response. See Appendix 5 for a full [illegible]

## 16-ii) Primary analysis should be intent-to-treat

Primary analysis should be intent-to-treat, secondary analyses could include comparing only "users", with the appropriate caveats that this is no longer a randomized sample (see 18-i).

| | 1 | 2 | 3 | 4 | 5 | |
|---|---|---|---|---|---|---|
| subitem not at all important | ○ | ○ | ○ | ○ | ◉ | essential |

## Does your paper address subitem 16-ii?

Copy and paste relevant sections from the manuscript (include quotes in quotation marks "like this" to indicate direct quotes from your manuscript), or elaborate on this item by providing additional information not in the ms, or briefly explain why the item is not applicable/relevant for your study

Primary analysis was intent-to-treat.

## 17a) For each primary and secondary outcome, results for each group, and the estimated effect size and its precision (such as 95% confidence interval)

## Does your paper address CONSORT subitem 17a? *

Copy and paste relevant sections from the manuscript (include quotes in quotation marks "like this" to indicate direct quotes from your manuscript), or elaborate on this item by providing additional information not in the ms, or briefly explain why the item is not applicable/relevant for your study

"After adjusting for time (period) and mean number of MET minutes at baseline, cluster-level linear regression yielded a non-statistically significant difference in the grand mean number of MET minutes reported per week at follow-up between intervention and control conditions (MD 1027, 95% CI -155 to 2209; p=.09)."
"Outliers were, on average, more likely to self-report a significantly greater number of MET minutes at baseline versus non-outliers (MD 5650, 95% CI 4082 to 7218); otherwise, the distribution of all other baseline characteristics was statistically equivalent between outliers and non-outliers. After excluding outliers, the subsequent linear regression yielded a non-statistically significant, and less positive (closer to the null), difference in the grand mean number of MET minutes reported per week between intervention and control conditions (MD 487, 95% CI -298 to 1273; p=.22).
"There were no significant treatment effects on action self-efficacy (n=392; MD (intervention-control) -1.73, 95% CI -5.56 to 2.11; p=.38), maintenance self-efficacy (n=361; MD (intervention-control) -1.92, 95% CI -5.68 to 1.85; p=.32), recovery self-efficacy (n=420; MD (intervention-control) 2.28, 95% CI -1.39 to 5.94; p=.22), and overall self-efficacy (n=413; MD (intervention-control) 1.13, 95% CI -1.73 to 4.00; p=.44). There were also no significant differences in the mean proportion of subjects who were in the volitional phase at 4 months (PR(intervention/control) 0.95, 95% CI 0.14 to 6.66; p=.96), or those who were classified as 'actors' at 4 months (PR(intervention/control) 0.88, 95% CI 0.11 [illegible]

## 17a-i) Presentation of process outcomes such as metrics of use and intensity of use

In addition to primary/secondary (clinical) outcomes, the presentation of process outcomes such as metrics of use and intensity of use (dose, exposure) and their operational definitions is critical. This does not only refer to metrics of attrition (13-b) (often a binary variable), but also to more continuous exposure metrics such as "average session length". These must be accompanied by a technical description how a metric like a "session" is defined (e.g., timeout after idle time) [1] (report under item 6a).

| | 1 | 2 | 3 | 4 | 5 | |
|---|---|---|---|---|---|---|
| subitem not at all important | ○ | ○ | ○ | ○ | ◉ | essential |

## Does your paper address subitem 17a-i?

Copy and paste relevant sections from the manuscript (include quotes in quotation marks "like this" to indicate direct quotes from your manuscript), or elaborate on this item by providing additional information not in the ms, or briefly explain why the item is not applicable/relevant for your study

These can be found in Appendix 5.

# 17b) For binary outcomes, presentation of both absolute and relative effect sizes is recommended

## Does your paper address CONSORT subitem 17b? *

Copy and paste relevant sections from the manuscript (include quotes in quotation marks "like this" to indicate direct quotes from your manuscript), or elaborate on this item by providing additional information not in the ms, or briefly explain why the item is not applicable/relevant for your study

N/A

# 18) Results of any other analyses performed, including subgroup analyses and adjusted analyses, distinguishing pre-specified from exploratory

## Does your paper address CONSORT subitem 18? *

Copy and paste relevant sections from the manuscript (include quotes in quotation marks "like this" to indicate direct quotes from your manuscript), or elaborate on this item by providing additional information not in the ms, or briefly explain why the item is not applicable/relevant for your study

"After adjusting for time (period) and mean number of MET minutes at baseline, cluster-level linear regression yielded a non-statistically significant difference in the grand mean number of MET minutes reported per week at follow-up between intervention and control conditions (MD 1027, 95% CI -155 to 2209; p=.09)."

## 18-i) Subgroup analysis of comparing only users

A subgroup analysis of comparing only users is not uncommon in ehealth trials, but if done, it must be stressed that this is a self-selected sample and no longer an unbiased sample from a randomized trial (see 16-iii).

| | 1 | 2 | 3 | 4 | 5 | |
|---|---|---|---|---|---|---|
| subitem not at all important | ○ | ○ | ○ | ○ | ● | essential |

## Does your paper address subitem 18-i?

Copy and paste relevant sections from the manuscript (include quotes in quotation marks "like this" to indicate direct quotes from your manuscript), or elaborate on this item by providing additional information not in the ms, or briefly explain why the item is not applicable/relevant for your study

"In total, 183/296 (61.8%) of patients exposed to the intervention handed in a process evaluation survey following their PHR. Only 112 completed the survey fully, which represents 61.2% of those who handed in a survey and only 37.8% of the intervention group. Overall, fewer than half of respondents (88/178 or 49.4%) stated they received at least a PA prescription from their PCP. A chi-square test of independence indicated no significant difference in the proportion of patients who received at least a PA prescription versus no materials between teams ($\chi 2(3)=3.03$; P-value=0.39). Among the 88 patients who received a PA prescription, just under half (42/88 or 47.7%) also received tailored resources to take home. The proportion of intervention patients who completed the process evaluation receiving both a PA prescription and resources ranged from a low of 9.4% (6/64 patients) for team 1 to a high of 45.5% (15/33 patients) for team 3.

Only 12/183 (6.6%) patients completing a process evaluation indicated that no PA discussion occurred during their appointment. Nearly half (86/176 or 48.9%) of participants who estimated the length of their PA discussion reported a length of 2-5 minutes, with patients in team 4 were more likely to report a talk of <2 minutes. Most patients reported being satisfied with their PA discussion irrespective of team, with no patients indicating they were dissatisfied. Of the process evaluation questions, patient satisfaction with their PA counselling (if applicable) was most prone to missing responses, with only 114/183 (62.3%) providing a response. See Appendix 5 for a full [illegible]

## 19) All important harms or unintended effects in each group

(for specific guidance see CONSORT for harms)

### Does your paper address CONSORT subitem 19? *

Copy and paste relevant sections from the manuscript (include quotes in quotation marks "like this" to indicate direct quotes from your manuscript), or elaborate on this item by providing additional information not in the ms, or briefly explain why the item is not applicable/relevant for your study

No harms/unintended effects to report.

### 19-i) Include privacy breaches, technical problems

Include privacy breaches, technical problems. This does not only include physical “harm” to participants, but also incidents such as perceived or real privacy breaches [1], technical problems, and other unexpected/unintended incidents. “Unintended effects” also includes unintended positive effects [2].

| | 1 | 2 | 3 | 4 | 5 | |
|---|---|---|---|---|---|---|
| subitem not at all important | ○ | ○ | ○ | ○ | ◉ | essential |

### Does your paper address subitem 19-i?

Copy and paste relevant sections from the manuscript (include quotes in quotation marks "like this" to indicate direct quotes from your manuscript), or elaborate on this item by providing additional information not in the ms, or briefly explain why the item is not applicable/relevant for your study

No privacy breaches or technical problems to report.

### 19-ii) Include qualitative feedback from participants or observations from staff/researchers

Include qualitative feedback from participants or observations from staff/researchers, if available, on strengths and shortcomings of the application, especially if they point to unintended/unexpected effects or uses. This includes (if available) reasons for why people did or did not use the application as intended by the developers.

| | 1 | 2 | 3 | 4 | 5 | |
|---|---|---|---|---|---|---|
| subitem not at all important | ○ | ○ | ○ | ○ | ◉ | essential |

### Does your paper address subitem 19-ii?

Copy and paste relevant sections from the manuscript (include quotes in quotation marks "like this" to indicate direct quotes from your manuscript), or elaborate on this item by providing additional information not in the ms, or briefly explain why the item is not applicable/relevant for your study

Qualitative feedback was not retrieved from participants or staff, other than the completion of the process evaluation by patient participants.

## DISCUSSION

## 22) Interpretation consistent with results, balancing benefits and harms, and considering other relevant evidence

NPT: In addition, take into account the choice of the comparator, lack of or partial blinding, and unequal expertise of care providers or centers in each group

### 22-i) Restate study questions and summarize the answers suggested by the data, starting with primary outcomes and process outcomes (use)

Restate study questions and summarize the answers suggested by the data, starting with primary outcomes and process outcomes (use).

| | 1 | 2 | 3 | 4 | 5 | |
|---|---|---|---|---|---|---|
| subitem not at all important | ○ | ○ | ○ | ○ | ◉ | essential |

## Does your paper address subitem 22-i? *

Copy and paste relevant sections from the manuscript (include quotes in quotation marks "like this" to indicate direct quotes from your manuscript), or elaborate on this item by providing additional information not in the ms, or briefly explain why the item is not applicable/relevant for your study

"This study assessed the feasibility of implementing a primary care-based eHealth tool to screen for PA levels and provide tailored, evidence-based resources for both providers and patients. Over the course of 6 months, 530 patients were enrolled, with limited investment in personnel. Results show a trend towards improvement in PA levels for those who received the intervention, although the unexpectedly high variability limited statistical power."

"The process evaluation indicates that almost all patients in the intervention arm received counselling about PA, almost half received a PA prescription and most were highly satisfied with counselling they received."

## 22-ii) Highlight unanswered new questions, suggest future research

Highlight unanswered new questions, suggest future research.

| | 1 | 2 | 3 | 4 | 5 | |
|---|---|---|---|---|---|---|
| subitem not at all important | ○ | ○ | ○ | ○ | ● | essential |

## Does your paper address subitem 22-ii?

Copy and paste relevant sections from the manuscript (include quotes in quotation marks "like this" to indicate direct quotes from your manuscript), or elaborate on this item by providing additional information not in the ms, or briefly explain why the item is not applicable/relevant for your study

"It is possible that sending tailored information directly to patients prior to an appointment may facilitate shared decision-making on PA during the clinical encounter [58,59]."
"In addition to careful consideration of outcome measures, appropriate patient selection is an important consideration for future work in this area. It is possible that patients who attend clinic for a PHR may be systematically different (i.e., biased toward an interest or willingness to engage in healthy lifestyle behaviours) from the general population. PHR visits were used as they present a highly feasible time to incorporate structured counselling on PA."
"As a SW-CRT design is suitable to test the effect of an intervention on PA, future studies must recruit a large number of clusters to minimize the aforementioned issues. This would enable use of more conventional, mixed-effects regression that accounts for clustering via random effects and involves a greater number of observations (via avoiding aggregation) that can result in more power to detect treatment effects, if truly present [65], and adjust for baseline imbalances with reduced concern of overfitting.  Further, our process evaluation had a high level of missing data, particularly on the overall satisfaction question, increasing the risk of bias in the reported results. It is possible that those who were not satisfied with the intervention were less likely to respond. Steps to increase response rates to process measure surveys, including electronic delivery, should be considered for future work."

---

## 20) Trial limitations, addressing sources of potential bias, imprecision, and, if relevant, multiplicity of analyses

### 20-i) Typical limitations in ehealth trials

Typical limitations in ehealth trials: Participants in ehealth trials are rarely blinded. Ehealth trials often look at a multiplicity of outcomes, increasing risk for a Type I error. Discuss biases due to non-use of the intervention/usability issues, biases through informed consent procedures, unexpected events.

| | 1 | 2 | 3 | 4 | 5 | |
|---|---|---|---|---|---|---|
| subitem not at all important | ○ | ○ | ○ | ○ | ◉ | essential |

### Does your paper address subitem 20-i? *

Copy and paste relevant sections from the manuscript (include quotes in quotation marks "like this" to indicate direct quotes from your manuscript), or elaborate on this item by providing additional information not in the ms, or briefly explain why the item is not applicable/relevant for your study

"Further, our process evaluation had a high level of missing data, particularly on the overall satisfaction question, increasing the risk of bias in the reported results. It is possible that those who were not satisfied with the intervention were less likely to respond. Steps to increase response rates to process measure surveys, including electronic delivery, should be considered for future work."

---

## 21) Generalisability (external validity, applicability) of the trial findings

NPT: External validity of the trial findings according to the intervention, comparators, patients, and care providers or centers involved in the trial

### 21-i) Generalizability to other populations

Generalizability to other populations: In particular, discuss generalizability to a general Internet population, outside of a RCT setting, and general patient population, including applicability of the study results for other organizations

| | 1 | 2 | 3 | 4 | 5 | |
|---|---|---|---|---|---|---|
| subitem not at all important | ○ | ○ | ○ | ○ | ◉ | essential |

## Does your paper address subitem 21-i?

Copy and paste relevant sections from the manuscript (include quotes in quotation marks "like this" to indicate direct quotes from your manuscript), or elaborate on this item by providing additional information not in the ms, or briefly explain why the item is not applicable/relevant for your study

"It is possible that patients who attend clinic for a PHR may be systematically different (i.e., biased toward an interest or willingness to engage in healthy lifestyle behaviours) from the general population. PHR visits were used as they present a highly feasible time to incorporate structured counselling on PA. However, this potential bias may explain why patients in our study had much higher than expected levels of PA. It is also possible that focusing on these types of visits limits the potential for effectiveness of the intervention if PA is already routinely discussed during usual care. Unfortunately, resources were not available to capture process data from usual care patients in this pilot trial."

## 21-ii) Discuss if there were elements in the RCT that would be different in a routine application setting

Discuss if there were elements in the RCT that would be different in a routine application setting (e.g., prompts/reminders, more human involvement, training sessions or other co-interventions) and what impact the omission of these elements could have on use, adoption, or outcomes if the intervention is applied outside of a RCT setting.

| | 1 | 2 | 3 | 4 | 5 | |
|---|---|---|---|---|---|---|
| subitem not at all important | ○ | ○ | ○ | ○ | ● | essential |

## Does your paper address subitem 21-ii?

Copy and paste relevant sections from the manuscript (include quotes in quotation marks "like this" to indicate direct quotes from your manuscript), or elaborate on this item by providing additional information not in the ms, or briefly explain why the item is not applicable/relevant for your study

This intervention was found to be acceptable for the Women's College Hospital Family Practice Health Centre. As such, most elements of this RCT can be implemented in routine setting, based on availability of resources (such as tablets and trained personnel that were available as a result of study funding).

## OTHER INFORMATION

## 23) Registration number and name of trial registry

### Does your paper address CONSORT subitem 23? *

Copy and paste relevant sections from the manuscript (include quotes in quotation marks "like this" to indicate direct quotes from your manuscript), or elaborate on this item by providing additional information not in the ms, or briefly explain why the item is not applicable/relevant for your study

"registered on ClinicalTrials.gov as NCT03181295"

## 24) Where the full trial protocol can be accessed, if available

### Does your paper address CONSORT subitem 24? *

Cite a Multimedia Appendix, other reference, or copy and paste relevant sections from the manuscript (include quotes in quotation marks "like this" to indicate direct quotes from your manuscript), or elaborate on this item by providing additional information not in the ms, or briefly explain why the item is not applicable/relevant for your study

The full trial protocol has not been published or included as an appendix. It is available upon request.

## 25) Sources of funding and other support (such as supply of drugs), role of funders

### Does your paper address CONSORT subitem 25? *

Copy and paste relevant sections from the manuscript (include quotes in quotation marks "like this" to indicate direct quotes from your manuscript), or elaborate on this item by providing additional information not in the ms, or briefly explain why the item is not applicable/relevant for your study

"This research was funded by the Academic Health Sciences Centre (AHSC) Alternative Funding Plan (AFP) Innovation Fund."

## X27) Conflicts of Interest (not a CONSORT item)

### X27-i) State the relation of the study team towards the system being evaluated

In addition to the usual declaration of interests (financial or otherwise), also state the relation of the study team towards the system being evaluated, i.e., state if the authors/evaluators are distinct from or identical with the developers/sponsors of the intervention.

| | 1 | 2 | 3 | 4 | 5 | |
|---|---|---|---|---|---|---|
| subitem not at all important | ○ | ○ | ○ | ○ | ◉ | essential |

### Does your paper address subitem X27-i?

Copy and paste relevant sections from the manuscript (include quotes in quotation marks "like this" to indicate direct quotes from your manuscript), or elaborate on this item by providing additional information not in the ms, or briefly explain why the item is not applicable/relevant for your study

N/A - this was not an industry-sponsored study.

## About the CONSORT EHEALTH checklist

### As a result of using this checklist, did you make changes in your manuscript? *

- ○ yes, major changes
- ◉ yes, minor changes
- ○ no

### What were the most important changes you made as a result of using this checklist?

Inclusion of minor details, potentially relevant to other researchers.

### How much time did you spend on going through the checklist INCLUDING making changes in your manuscript *

2 days

As a result of using this checklist, do you think your manuscript has improved? *

- (x) yes
- ( ) no
- ( ) Other:

Would you like to become involved in the CONSORT EHEALTH group?

This would involve for example becoming involved in participating in a workshop and writing an "Explanation and Elaboration" document

- ( ) yes
- (x) no
- ( ) Other:

Any other comments or questions on CONSORT EHEALTH

Your answer

## STOP - Save this form as PDF before you click submit

To generate a record that you filled in this form, we recommend to generate a PDF of this page (on a Mac, simply select "print" and then select "print as PDF") before you submit it.

When you submit your (revised) paper to JMIR, please upload the PDF as supplementary file.

Don't worry if some text in the textboxes is cut off, as we still have the complete information in our database. Thank you!

## Final step: Click submit !

Click submit so we have your answers in our database!

SUBMIT

Never submit passwords through Google Forms.

This content is neither created nor endorsed by Google. Report Abuse - Terms of Service

Google Forms
